# Developing and validating a clinlabomics-based machine-learning model for early detection of retinal detachment in patients with high myopia

**DOI:** 10.1186/s12967-024-05131-9

**Published:** 2024-04-30

**Authors:** Shengjie Li, Meiyan Li, Jianing Wu, Yingzhu Li, Jianping Han, Yunxiao Song, Wenjun Cao, Xingtao Zhou

**Affiliations:** 1 Department of Clinical Laboratory, Eye & ENT Hospital, Shanghai Medical College, Fudan University, Shanghai, China; 2 Department of Ophthalmology and Optometry, Fudan University Eye Ear Nose and Throat Hospital, Shanghai, China; 3 NHC Key Laboratory of Myopia (Fudan University), Shanghai, China; 4 Key Laboratory of Myopia, Chinese Academy of Medical Sciences, Shanghai, 200031 China; 5 Shanghai Research Center of Ophthalmology and Optometry, Shanghai, China; 6 Shanghai Engineering Research Center of Laser and Autostereoscopic 3D for Vision Care, Shanghai, China; 7 Department of Clinical Laboratory, Shanghai Xuhui Central Hospital, Fudan University, Shanghai, China

**Keywords:** Retinal detachment, High myopia, Clinlabomics, Machine-learning, Detection

## Abstract

**Background:**

Retinal detachment (RD) is a vision-threatening disorder of significant severity. Individuals with high myopia (HM) face a 2 to 6 times higher risk of developing RD compared to non-myopes. The timely identification of high myopia-related retinal detachment (HMRD) is crucial for effective treatment and prevention of additional vision impairment. Consequently, our objective was to streamline and validate a machine-learning model based on clinical laboratory omics (clinlabomics) for the early detection of RD in HM patients.

**Methods:**

We extracted clinlabomics data from the electronic health records for 24,440 HM and 5607 HMRD between 2015 and 2022. Lasso regression analysis assessed fifty-nine variables, excluding collinear variables (variance inflation factor > 10). Four models based on random forest, gradient boosting machine (GBM), generalized linear model, and Deep Learning Model were trained for HMRD diagnosis and employed for internal validation. An external test of the models was done. Three random data sets were further processed to validate the performance of the diagnostic model. The primary outcomes were the area under the receiver operating characteristic curve (AUC) and the area under the precision-recall curve (AUCPR) to diagnose HMRD.

**Results:**

Nine variables were selected by all models. Given the AUC and AUCPR values across the different sets, the GBM model was chosen as the final diagnostic model. The GBM model had an AUC of 0.8550 (95%CI = 0.8322–0.8967) and an AUCPR of 0.5584 (95%CI = 0.5250–0.5879) in the training set. The AUC and AUCPR in the internal validation were 0.8405 (95%CI = 0.8060–0.8966) and 0.5355 (95%CI = 0.4988–0.5732). During the external test evaluation, it reached an AUC of 0.7579 (95%CI = 0.7340–0.7840) and an AUCPR of 0.5587 (95%CI = 0.5345–0.5880). A similar discriminative capacity was observed in the three random data sets. The GBM model was well-calibrated across all the sets. The GBM-RD model was implemented into a web application that provides risk prediction for HM individuals.

**Conclusion:**

GBM algorithms based on nine features successfully predicted the diagnosis of RD in patients with HM, which will help ophthalmologists to establish a preliminary diagnosis and to improve diagnostic accuracy in the clinic.

**Supplementary Information:**

The online version contains supplementary material available at 10.1186/s12967-024-05131-9.

## Introduction

Retinal detachment (RD) is a severe vision-threatening disorder that separates the neurosensory retina from the underlying retinal pigment epithelium [1]. The annual incidence of RD ranged from 6.9 [2] to 22.0 [3] cases per 100 000 persons, with an increasing average yearly incidence rate [4]. Numerous risk factors have been linked to RD, encompassing the process of aging, myopia, severe ocular trauma, prior ocular surgeries such as cataract extraction, and ocular conditions such as lattice degeneration [1]. By 2050, it is projected that 50% of people worldwide will have myopia, and vital epidemiologic data link myopia with RD [5]. Each additional diopter (D) of myopia is associated with a 30% increase in the risk of RD [6, 7], high myopia (HM) individuals are 2 to 6 times more likely to get RD than non-myopes people [8]. Early RD identification is crucial to slow down or stop the growth of this chronic, blind-threatening condition.

Up to now, RD predicting diagnosis in HM eyes relies on professional ophthalmologists and ophthalmic equipment. Risk prediction or diagnostic prediction is crucial to assess eligibility for surgery. In addition, this information can assist patients and ophthalmologists in collaborative decision-making processes that direct therapy. Mixing medicine with machine learning algorithms has developed into a potent instrument for changing health care, including the nature of illness screening in clinical diagnosis, which was also proved in ophthalmology. Several fundus image-based models for RD detection have been developed [9–12], all based on a deep learning algorithm and using the fundus image. Even though these fundus image-based models performed better, their reliance on specialized eye examination tools. Notably, individuals do not often see an ophthalmologist until their symptoms worsen or their vision suddenly deteriorates in China. Consequently, using only fundus image-based models makes detecting and diagnosing high myopia with retinal detachment (HMRD) early on difficult. Thus, there is still a clinical need to create a quick, accurate, and practical screening method to find HMRD.

Clinical laboratory medicine and machine learning algorithms have been combined to create a new concept of clinical laboratory omics (Clinlabomics), which uses high-throughput methods to extract significant amounts of feature data from blood, bodily fluids, secretions, excreta, and cast clinical laboratory test data [13]. Clinlabomics-based deep-learning algorithms have been successfully applied to various diseases in recent years [14–16]. For example, Schneider et al. [15] validated a prediction model produced by a machine-learning algorithm that used complete blood cell count to identify those who were more likely to develop colorectal cancer. However, few studies have developed predicting diagnosis algorithms based on Clinlabomics to identify eye diseases.

The pathophysiology of RD is thought to involve several pathogenic processes, including inflammation [17], blood circulation disorders [18], and metabolic disturbances [19]. Routine blood indices, biochemical indices, and coagulation indices can reflect a wide range of physiological and pathological states in the body [20], providing information on aspects such as inflammation, blood circulation, metabolic status, and tissue injury. Research indicates that blood markers of inflammation [21], glucose levels [22], and lipid levels [23] are associated with an increased risk of ocular diseases, including HM, and RD. Our previous study developed a routine blood parameters-based model for serial monitoring and predicting the occurrence of RD in HM [24]. While innovative at the time, this model was constrained by its dependence on a relatively narrow set of blood indices (*n* = 22), exhibiting moderate performance with an area under the curve (AUC) of approximately 0.77–0.81. Critically, it lacked validation through external testing.

Thus, the current study utilized Clinlabomics data (routine blood indices, biochemical indices, and coagulation indices) from two centers, combined with machine learning methods, to develop a clinically useful screening model for RD in HM individuals. This was followed by both internal and external validation of the model.

## Materials and methods

### Study design and population

In this retrospective two-center study, we developed and validated four models (Random Forest (RF), Gradient Boosting Machine [GBM], Generalized Linear Model [GLM], and Deep Learning Model) for screening RD in patients with HM using demographic data and clinical laboratory omics (Clinlabomics) data from two hospitals. This study was conducted following the principles of “Transparent Reporting of a Multivariable Prediction Model for Individual Prognosis or Diagnosis (TRIPOD) [25] ”. This study was approved by the Ethics Committee of Eye and ENT Hospital of Fudan University (EENT-2,015,011) and was conducted under the Declaration of Helsinki. All participants provided written informed consent prior to their participation. All patients underwent a comprehensive ophthalmologic examination as described previously [26–28] and detailed in the supplementary material. The inclusion and exclusion criteria were described previously [24] and detailed in the supplementary material.

A total of 23,778 patients with HM and 5432 patients with HMRD were recruited from the Eye and ENT Hospital of Fudan University, Shanghai, China, from June 2015 to December 2022. Following the inclusion and exclusion criteria as described, 360 patients (HM = 205, HMRD = 155) and 805 patients (HM = 620, HMRD = 185) were excluded, respectively. Finally, a total of 22,953 patients with HM and 5092 patients with HMRD were included.

For the external test cohort (*n* = 2179, from January 2017 to December 2022), patients diagnosed with HM and HMRD were recruited from Shanghai Xuhui Central Hospital, Shanghai, China. After applying the inclusion and exclusion criteria as previously described, 1487 patients with HM and 515 patients with HMRD were included, while 56 patients (HM = 41, HMRD = 15) and 121 patients (HM = 96, HMRD = 25) were excluded.

In total, 24,440 HM and 5607 HMRD visits in the Eye and ENT Hospital of Fudan University and Xuhui Central hospital between 2015 and 2022 were included.

### Data sources

For this multi-institutional cohort study, data were retrieved from the electronic medical record. The electronic medical record included demographic data and Clinlabomics data. The principal investigator at each institution collects fifty-nine variables from each patient. The Clinlabomics dataset consists of blood cell analysis [twenty-four variables: neutrophil, neutrophil%, red blood count (RBC), thrombocytocrit (PCT), platelet count (PLT), platelet distribution width (PDW), hemoglobin (HG), eosinophil, eosinophil%, basophil, basophil%, mean platelet volume (MPV), lymphocyte, lymphocyte%, hematokrit (HCT), monocyte, monocyte%, platelet large cell ratio (PLCR), white blood cell count (WBC), red blood cell distribution width-standard deviation (RBCSD), red blood cell distribution width- coefficient of variation (RBCCV), mean corpuscular volume (MCV), mean corpuscular hemoglobin concentration (MCHC), and mean corpuscular hemoglobin (MCH)], biochemistry analysis [twenty-six variables: total protein (TP), prealbumin (PAB), total bile acid (TBA), total bilirubin (TBIL), total cholesterol (TC), albumin (ALB), AG, glucose (GLU), lactic dehydrogenase (LDH), globulin (GLB), uric acid (UA), blood urea nitrogen (BUN), direct bilirubin (DBIL), alkaline phosphatase (ALP), creatine kinase (CK), creatinine (CREA), glutamic oxalacetic transaminase (AST), glutamic-pyruvic transaminase (ALT), gamma-glutamyl transpeptidase (GGT), triglyceride (TG), potassium (K), SODIUM, chloridion (CL), phosphorus (P), calcium (Ca), glycosylated hemoglobin (HbA1c)], and blood coagulation analysis [Seven variables: fibrinogen (FIB), prothrombin time (PT), thrombin time (TT), activated partial thromboplastin time (APTT), international normalized ratio (INR), PT%, and d-dimer (DD)]. Laboratory tests were performed at the time of the RD occurrence.

### Blood cell analysis

In the morning, after 8 h of fasting, 2 mL of blood samples were drawn from the participants’ antecubital fossae (anterior elbow veins) through standard venipuncture. The samples were collected in ethylenediaminetetraacetic acid tubes and tested within 0.5 h in the Department of Clinical Laboratory of Eye and ENT Hospital of Fudan University (Sysmex series automated blood counting system, Kobe, Japan) and the Department of Clinical Laboratory of Shanghai Xuhui Central Hospital (Mindray series automated blood counting system, Shenzhen, China).

### Biochemistry analysis

After an 8-hour fast, blood samples were collected via standard venipuncture from the antecubital fossae (anterior elbow veins). All sample tubes were centrifuged at 3,000 rpm for 10 min, and all serum samples were tested within 3 h. Laboratory tests were conducted at the Department of Clinical Laboratory of Eye and ENT Hospital of Fudan University (Cobs 702, Roche Diagnostics GmbH, Mannheim, Germany) and the Department of Clinical Laboratory of Shanghai Xuhui Central Hospital (BS-2000M2, Mindray automatic biochemical analyzer, Shenzhen, China).

### Blood coagulation analysis

In the morning, after 8 h of fasting, 3 ml of blood samples were drawn from the participants’ antecubital fossae (anterior elbow veins) through standard venipuncture. The samples were collected in sodium citrate anticoagulation tubes and tested within 3 h in the Department of Clinical Laboratory of Eye and ENT Hospital of Fudan University (STAGO STA-R Evolution, France) and the Department of Clinical Laboratory of Shanghai Xuhui Central Hospital (EXC810, Mindray automatic coagulation analyzer, Shenzhen, China).

## Model development and validate

**Fig. 1:**
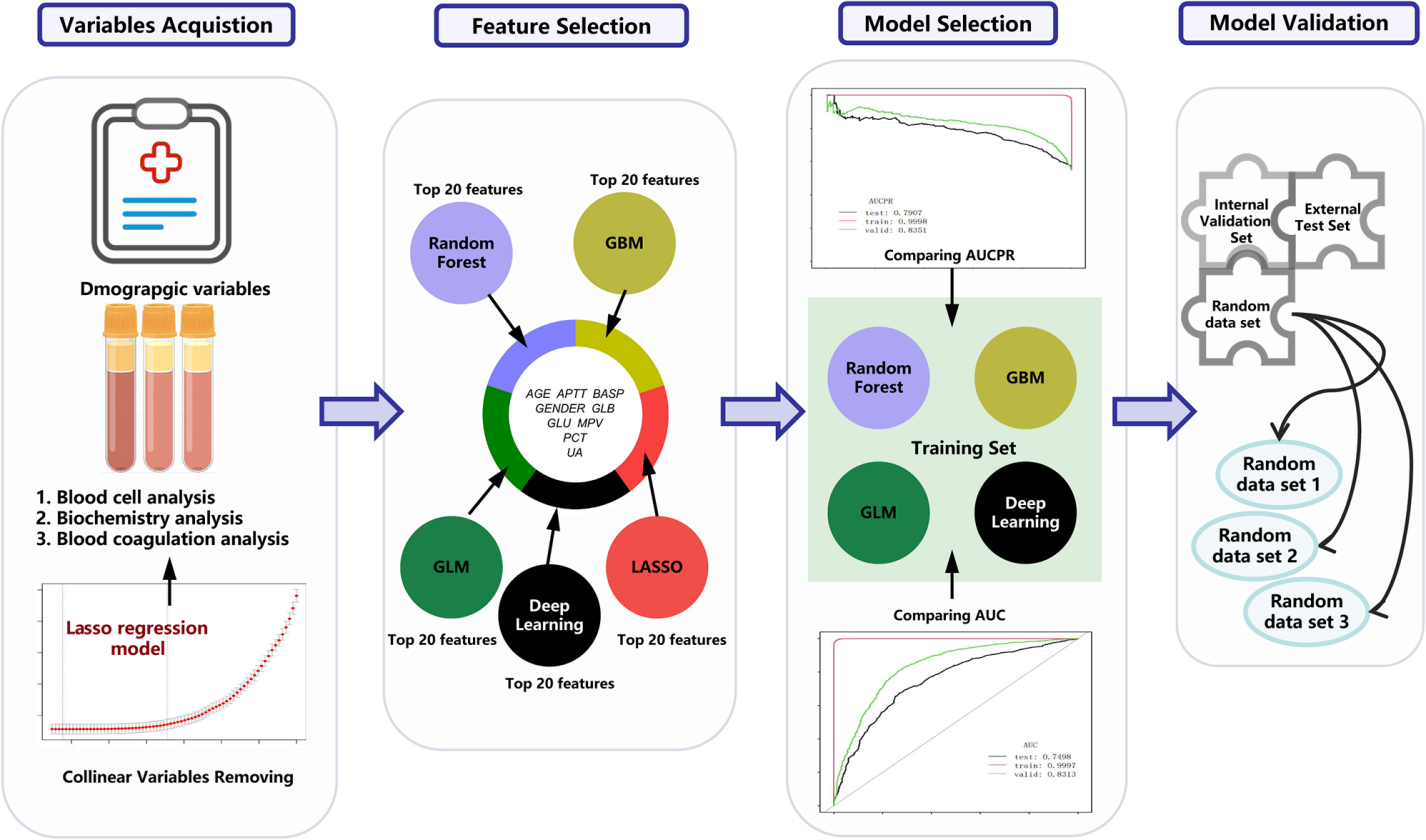
Study flow-chart: This figure displays the participant flow-chart. GLU: glucose; PCT: thrombocytocrit; MPV: mean platelet volume; UA: uric acid; APTT: activated partial thromboplastin time; GLB: globulin; BASP: percentage of basophil. GBM: gradient boosting machine; GLM: generalized linear model

The development of the model consisted of four main stages (Fig. 1): (1) variables acquisition; (2) feature selection; (3) model selection; and (4) model validation.

### Variables acquisition

We selected laboratory tests measured in more than 90% of the patients as diagnostic variables. All demographic and Clinlabomics variables available have been used in the model and any variable selection method was used during the training process. Age, neutrophil, neutrophil%, RBC, PCT, PLT, PDW, HG, eosinophil, eosinophil%, basophil, basophil%, MPV, lymphocyte, lymphocyte%, HCT, monocyte, monocyte%, PLCR, WBC, RBCSD, RBCCV, MCV, MCHC, MCH, TP, PAB, TBA, TBIL, TC, ALB, AG, GLU, LDH, GLB, UA, BUN, DBIL, ALP, CK, CREA, AST, ALT, GGT, TG, K, SODIUM, CL, P, Ca, HbA1c, FIB, PT, TT, APTT, INR, PT%, and DD were considered as continuous variables. Gender was categorized as dichotomous variables. Missing values were imputed using mean-value.

### Feature selection

The candidate variable selection for the machine learning model was guided by our aim to simplify the model and was based on the training cohort.

First, the collinearity test was checked by running a collinearity diagnostic, which was built using the glmnet package in R software (https://www.r-project.org). Variance inflation factor (VIF) analysis was used to analyze the collinearity of fifty-nine variables, and the most colinear factor was deleted until no collinearity existed. Fifteen variables (AG, eosinophil, basophil HCT, HG, lymphocyte%, MCH, monocyte, neutrophil%, PLCR, PLT, PT, RBCCV, TP, WBC) were excluded owing to collinearity existed (VIF > 10). Forty-five variables were initially included to perform further analysis.

**Fig. 2:**
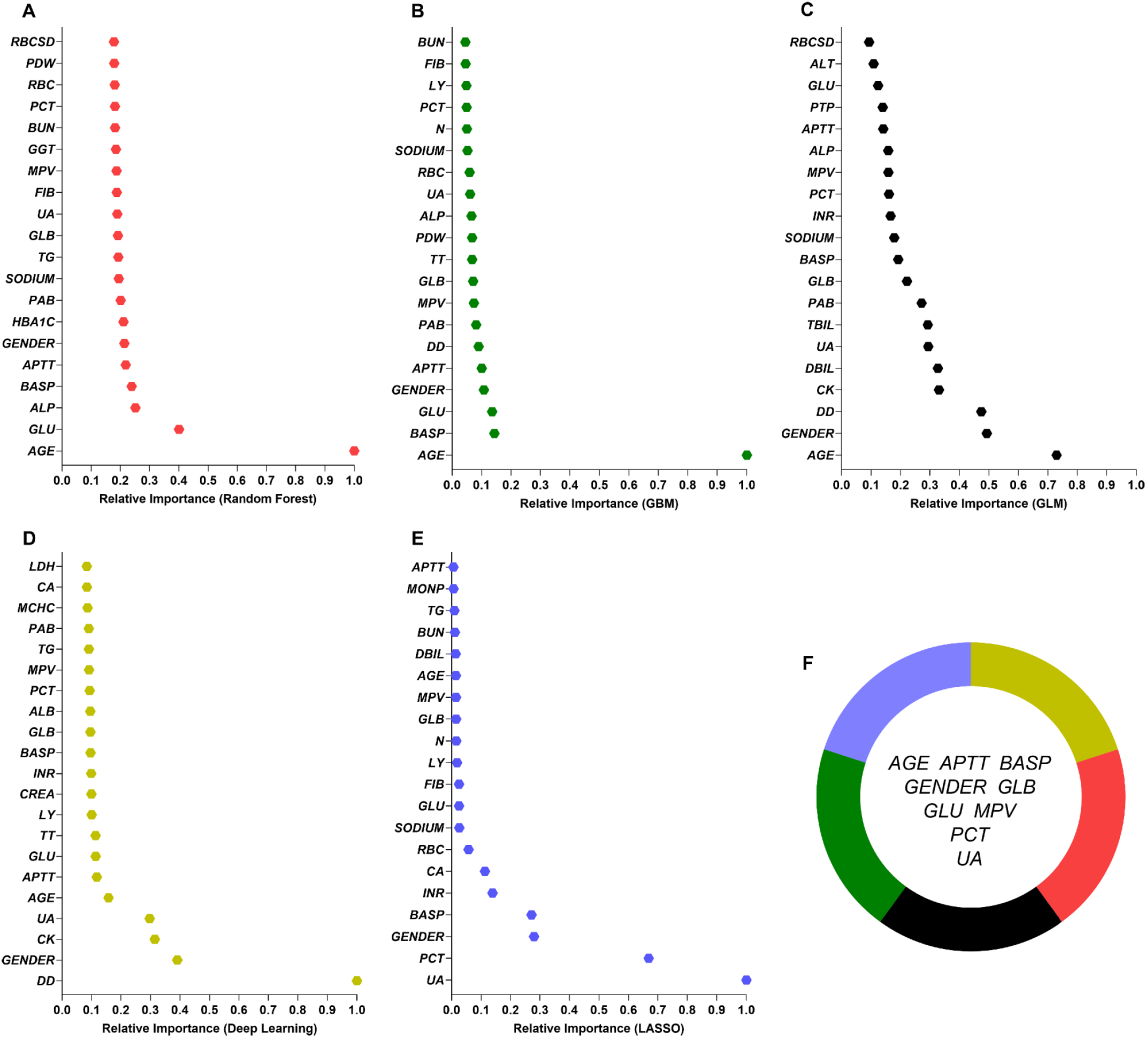
The top 20 significant variables chosen by five machine learning models (**A-E**) and the intersection set of these variables (**F**). TBA: total bile acid; TBIL: total bilirubin; TC: total cholesterol; N: neutrophil number; ALB: albumin; RBC: red blood count; HBA1C: glycosylated hemoglobin; PCT: thrombocytocrit; PDW: platelet distribution width; GLU: glucose; FIB: fibrinogen; EOSP: percentage of eosinophils; GLB: globulin; PAB: prealbumin; MPV: mean platelet volume; TT: thrombin time; UA: uric acid; BUN: blood urea nitrogen; P: phosphorus; LY: lymphocyte count; ALP: alkaline phosphatase; K: kalium; CK: creatine kinase; CREA: creatinine; AST: glutamic oxalacetic transaminase; ALT: glutamic-pyruvic transaminase; GGT: gamma-glutamyl transpeptidase; TG: triglyceride; CA: calcium; MONP: percentage of monocyte; APTT: activated partial thromboplastin time; INR: international normalized ratio; RBCSD: red blood cell distribution width-standard deviation; PTP: percentage of prothrombin time; MCV: mean corpuscular volume; MCHC: mean corpuscular hemoglobin concentration; DD: d-dimer; BASP: percentage of basophil; CL: chloridion; DBIL: direct bilirubin

Second, five different models (LASSO regression, RF, GBM, GLM, and Deep learning) were established to select the variables. The top 20 essential variables selected by the five models are shown in Fig. 2A-E. Finally, we chose the intersection set of these variables. Nine variables (Fig. 2F) were finally selected (age, APTT, BASP, gender, GLB, GLU, MPV, PCT, and UA).

### Model selection

The EENT dataset was established based on 23,778 patients with HM and 5432 patients with HMRD, randomly split into a training set (75%) and an internal validation set (25%). The diagnostic model of HMRD was established with the training dataset.

We conducted two sets of experiments. In the first experiment, all fifty-nine variables were pooled into four models (RF, GBM, GLM, and Deep learning) to develop a preliminary diagnostic model. In the second experiment, the selected nine variables were pooled into four models (RF classification, GBM, GLM, and deep learning) to develop a diagnostic model. Discrimination of the models was assessed using the AUC and the area under the receiver operating characteristic precision-recall curve (AUCPR). The most valuable model was obtained based on sensitivity, specificity, accuracy, AUC, AUCPR, positive predict value (PPV), negative predict value (NPV), and balanced accuracy of diagnostic indices.

### Model validation

We conducted three sets (internal validation set, external test set, and random data set) of experiments. First, an internal validation dataset was established and applied to validate the diagnostic efficacy of four models (RF, GBM, GLM, and deep learning) to diagnose HMRD. To evaluate the diagnostic potency, the sensitivity, specificity, accuracy, AUC, AUCPR, PPV, NPV, and balanced accuracy were computed with the h2o package in R software (https://www.r-project.org).

In total, 1487 patients with HM and 515 patients with HMRD admitted to the Shanghai Xuhui Central Hospital, Shanghai, were included as the external validation set. We used the selected diagnostic model to diagnose the probability of RD in HM patients. To evaluate the diagnostic potency, the sensitivity, specificity, accuracy, AUC, AUCPR, PPV, NPV, and balanced accuracy were computed with the h2o package in R software (https://www.r-project.org).

The random dataset was applied for model testing to address the class-imbalance problem, which could lead to a severely imbalanced degree of performance. In the random data set, two groups (RD, HMRD) of roughly equal size were randomly selected from RD and HM patients, respectively, and this procedure was repeated three times. We used the selected diagnostic model to diagnose the probability of RD in HM patients. To evaluate the diagnostic potency, the sensitivity, specificity, accuracy, AUC, AUCPR, PPV, NPV, and balanced accuracy were computed with the h2o package in R software (https://www.r-project.org). The calibration curve was also used to evaluate the performance of the final model.

### Sample size

To determine the minimum total sample size, an open-source calculator utilizing the methods described by Obuchowski et al. [29] and Li, et al. [30] was employed. The input parameters were specificity = 0.8 (allowable error = 0.05), sensitivity = 0.8 (allowable error = 0.05), and α = 0.025 (2-tailed). According to this calculation, the minimum sample size required for the new model development was 247 per group, while the total sample size in all our cohorts was at least two times higher than this minimum.

### Statistical analysis

We conducted descriptive statistical analyses for all variables, and normality was examined by the Shapiro–Wilk test. The difference between cases and controls was analyzed using multiple tests, such as an independent Student’s t-test for normally distributed continuous variables, the Kruskal-Wallis test for non-normally distributed continuous variables, and the Chi-squared test for categorical variables when necessary. Continuous variables were expressed as mean ± SD, and categorical variables were summarized as count and percentage. Pearson analysis was performed to analysis the relationship among age and other factors.

The Area Under the Precision-Recall Curve (AUCPR) and the Area Under the Receiver Operating Characteristics (AUC) curves were used to evaluate the discriminatory performances. The low prevalence of RD in individuals with HM indicates that the AUCPR is more resistant to class imbalances [31]. Calibration plots were used to visually evaluate the model calibration. A P-value of less than 0.05 was considered significant for all results.

All statistical analyses were performed using R software (http://www.R-project.org) and Empower Stats software (www.empowerstats.com), with parameters set to their default values.

## Results

### Cohort description

This two-center development and validation study used retrospective data from two hospitals where patients with RD or HMRD. Detailed information about the diagnostic variables of training, internal validation, and external testing datasets is presented in Table S1-S3. In this study, the average age of HM diagnosis is 24 years (range, 17–40). Most characteristics significantly differed among the training, internal validation, and external testing datasets. Table S4-S7 shows the diagnostic variables difference between HMRD and HM groups in training, internal validation, and external testing datasets. Most characteristics were significantly different between the HMRD and HM patients. The HMRD patients were more likely to be older (*P* < 0.05) than the HM patients. The Clinlabomics indexes were significantly different between the HMRD patients and HM patients. For example, the GLU, GLB, MPV, and UA level was higher in the HMRD patients than the HM patients.

### Development of the diagnostic model based on all features

Before creating the model, collinear variables were eliminated using the deviance residuals and the Lasso regression analysis. To begin with, 45 factors were added for additional study. Then, four models were established based on RF, GBM, GLM, and deep learning classification.

**Fig. 3:**
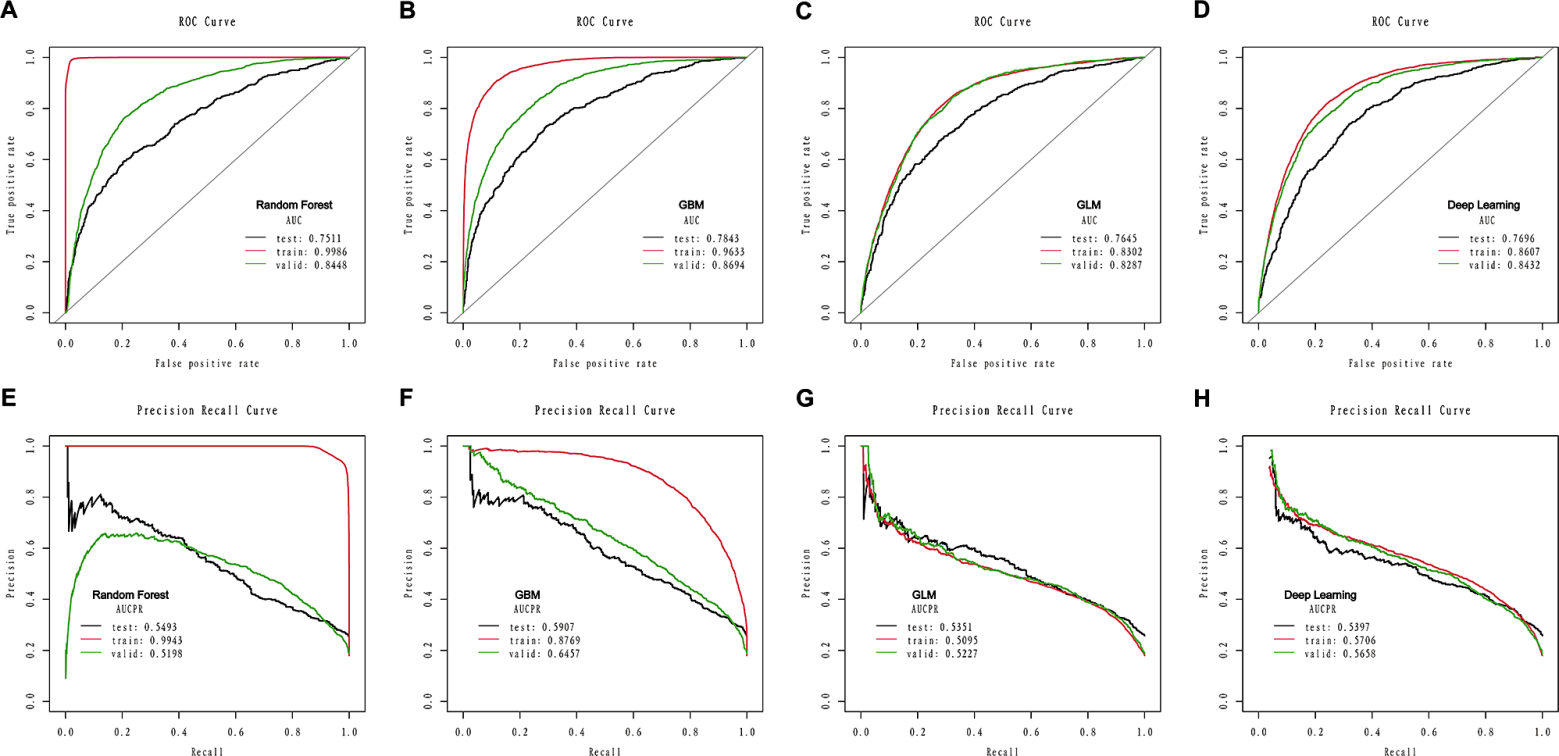
The area under the receiver operating characteristic curve (AUC) of the random forest (**A**), GBM (**B**), GLM (**C**), and deep learning (**D**) models based on all the variables in the training set, internal validation set and the external test set. The area under the precision-recall curve (AUCPR) of the random forest (**E**), GBM (**F**), GLM (**G**), and deep learning (**H**) models based on all the variables in the training set, internal validation set and the external test set

Based on the AUC and AUCPR, the RF and GBM models outperformed the GLM and deep learning models. A detailed description of the four models’ performance can be found in Table 1; Fig. 3. The RF model reached an AUC of 0.9986 and an AUCPR of 0.9943 during the training phase, visualized in Fig. 3A and E. The GBM model reached an AUC of 0.9633 and an AUCPR of 0.8769 during the training phase, visualized in Fig. 3B and F.

**Table 1:** Comparison of model performance, including all the variables on the train, internal validation, and external test set

Model	Sensitivity	Specificity	Accuracy	AUC (95% CI)	AUCPR (95% CI)	PPV	NPV	Balanced accuracy
Train								
RF	0.9823	0.9837	0.9835	0.9986 (0.9464-1.0)	0.9943 (0.9655-1.0)	0.9296	0.9961	0.9830
GBM	0.8562	0.9217	0.9099	0.9633 (0.9321-1.0)	0.8769 (0.8433–0.9125)	0.7051	0.9670	0.8889
GLM	0.6956	0.8026	0.7834	0.8302 (0.7825–0.9021)	0.5095 (0.4523–0.5667)	0.4353	0.9234	0.7491
DL	0.7007	0.8429	0.8174	0.8607 (0.8220–0.9005)	0.5706 (0.5245–0.6211)	0.4938	0.9279	0.7718
Validate								
RF	0.6409	0.8667	0.8243	0.8448 (0.7882–0.8990)	0.5198 (0.4878–0.5469)	0.5265	0.9125	0.7538
GBM	0.6469	0.8855	0.8407	0.8694 (0.8210–0.9106)	0.6457 (0.6122–0.6890)	0.5665	0.9156	0.7662
GLM	0.6925	0.8084	0.7866	0.8287 (0.7235–0.9207)	0.5227 (0.4655–0.5821)	0.4553	0.9191	0.7504
DL	0.6750	0.8432	0.8116	0.8432 (0.7109–0.8907)	0.5658 (0.5245–0.5929)	0.4989	0.9181	0.7591
Test								
RF	0.6291	0.7512	0.7198	0.7511 (0.7122–0.7957)	0.5493 (0.5126–0.5778)	0.4669	0.8540	0.6902
GBM	0.4291	0.9159	0.7907	0.7843 (0.7459–0.8202)	0.5907 (0.5691–0.6233)	0.6387	0.8225	0.6725
GLM	0.5612	0.8191	0.7527	0.7645 (0.7108–0.8056)	0.5351 (0.4789–0.5887)	0.5179	0.8435	0.6901
DL	0.8117	0.5958	0.6513	0.7696 (0.7245–0.7988)	0.5397 (0.4988–0.5887)	0.4102	0.9013	0.7037

### Internal-external validation based on all features

We validated the performance on the internal validation set and external test datasets from Shanghai Xuhui Central Hospital (Detailed in Table 1). Based on the AUC and AUCPR, the RF and GBM models outperformed the GLM and Deep learning models. Figure 3A shows that the RF model achieves an AUC of 0.8448 within the internal validation set and 0.7511 in the external test set. Figure 3E shows that the RF model achieves an AUCPR of 0.5198 within the internal validation set and an AUCPR of 0.5493 in the external test set. Figure 3B shows that the GBM model achieves an AUC of 0.8694 within the internal validation set and retains an AUC of 0.7843 in the external test set. Figure 3F shows that the GBM model achieves an AUCPR of 0.6457 within the internal validation set and retains an AUCPR of 0.5907 in the external test set. Overall, the GBM model showed the best discrimination capacity in the internal validation set and external test set.

### Features and feature importance

Based on the training database, LASSO regression, RF, GBM, GLM, and deep learning were established to select the variables. Figure 2 shows the twenty most essential features in the RF (Fig. 2A), GBM (Fig. 2B), GLM (Fig. 2C), deep learning (Fig. 2D), and LASSO regression (Fig. 2E) model in descending order. Next, we chose the intersection set of these variables. Nine variables (Fig. 2F) were finally selected (age, APTT, BASP, gender, GLB, GLU, MPV, PCT, and UA). There was no relationship among age, APTT, BASP, gender, GLB, GLU, MPV, PCT, and UA (*P* > 0.05), except UA and gender (Figure S1).

### Development of the diagnostic model based on nine features

**Fig. 4:**
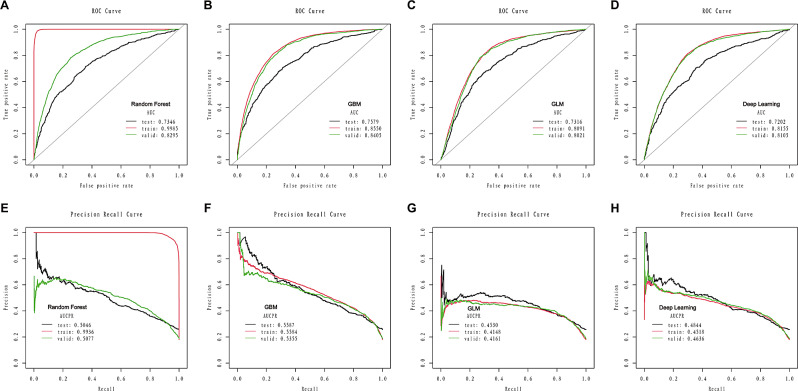
The area under the receiver operating characteristic curve (AUC) of the random forest (**A**), GBM (**B**), GLM (**C**), and deep learning (**D**) models based on the nine selected variables in the training set, internal validation set and the external test set. The area under the precision-recall curve (AUCPR) of the random forest (**E**), GBM (**F**), GLM (**G**), and deep learning (**H**) models based on the nine selected variables in the training set, internal validation set and the external test set

Based on the AUC and AUCPR, the RF and GBM models outperformed the GLM and deep learning models. A detailed description of the four models’ performance can be found in Table 2; Fig. 4. The RF model reached an AUC of 0.9985 and an AUCPR of 0.9936 during the training set, visualized in Fig. 4A and E. The GBM model achieved an AUC of 0.8550 and an AUCPR of 0.5584 during the training set, visualized in Fig. 4B and F.

**Table 2:** Comparison of model performance, including the selected variables on the train, internal validation, and external test set

Model	Sensitivity	Specificity	Accuracy	AUC (95% CI)	AUCPR (95% CI)	PPV	NPV	Balanced accuracy
Train								
RF	0.9902	0.9715	0.9749	0.9985 (0.9755-1.0)	0.9936 (0.9631-1.0)	0.8837	0.9978	0.9808
GBM	0.7425	0.8045	0.7934	0.8550 (0.8322–0.8967)	0.5584 (0.5250–0.5879)	0.4538	0.9346	0.7735
GLM	0.7706	0.7281	0.7358	0.8091 (0.7821–0.8394)	0.4148 (0.3567–0.4688)	0.3827	0.9355	0.7494
DL	0.7298	0.7603	0.7548	0.8155 (0.7655–0.8730)	0.4518 (0.4109–0.4890)	0.3997	0.9279	0.7451
Validate								
RF	0.6515	0.8435	0.8074	0.8295 (0.7682–0.8867)	0.5077 (0.4221–0.5781)	0.4906	0.9128	0.7475
GBM	0.7206	0.8030	0.7875	0.8405 (0.8060–0.8966)	0.5355 (0.4988–0.5732)	0.4582	0.9255	0.7618
GLM	0.7737	0.7359	0.7430	0.8021 (0.7544–0.8583)	0.4161 (0.3659–0.4765)	0.4039	0.9336	0.7548
DL	0.7221	0.7645	0.7565	0.8105 (0.7689–0.9763)	0.4636 (0.4356–0.4988)	0.4149	0.9224	0.7433
Test								
RF	0.5340	0.7989	0.7308	0.7346 (0.6875–0.7860)	0.5046 (0.4765–0.5467)	0.4791	0.8319	0.6665
GBM	0.2699	0.9496	0.7747	0.7579 (0.7340–0.7840)	0.5587 (0.5345–0.5880)	0.6495	0.7897	0.6097
GLM	0.6699	0.6987	0.6913	0.7316 (0.6937–0.7762)	0.4530 (0.4125–0.4990)	0.4351	0.8594	0.6843
DL	0.5379	0.7821	0.7193	0.7202 (0.6872–0.7688)	0.4844 (0.4221–0.5432)	0.4609	0.8301	0.6600

### Internal-external validation based on nine features

In the internal validation set (Table 2), the AUC for RF (Fig. 4A), GBM (Fig. 4B), GLM (Fig. 4C), and deep learning (Fig. 4D) models was 0.8295, 0.8405, 0.8021, and 0.8105, respectively. The AUCPR for RF (Fig. 4E), GBM (Fig. 4F), GLM (Fig. 4G), and deep learning (Fig. 4H) models was 0.5077, 0.5355, 0.4161, and 0.4636, respectively.

In the external test set (Table 2), the AUC for RF (Fig. 4A), GBM (Fig. 4B), GLM (Fig. 4C), and deep learning (Fig. 4D) models was 0.7346, 0.7579, 0.7316, and 0.7202, respectively. The AUCPR for RF (Fig. 4E), GBM (Fig. 4F), GLM (Fig. 4G), and Deep Learning (Fig. 4H) models was 0.5046, 0.5587, 0.4530, and 0.4844, respectively.

Overall, the GBM model showed the best discrimination capacity in the internal validation set and external test set. In addition, similar discriminative capacity was observed in the all-features-based GBM and nine-features-based GBM models.

### Random set evaluation

To avoid an over-fitting to imbalanced data, three random data sets were further processed to validate the performance of the diagnostic model. Similar results were also observed. Based on the AUC and AUCPR, in the train set, the RF model and GBM model outperformed the GLM and deep learning model in the random sampling 1 set (table S8, Figure S2), random sampling 2 set (table S9, Figure S3), and random sampling 3 set (table S9, Figure S4).

Meanwhile, the GBM model showed the best discrimination capacity in the internal validation set and external test set across the random sampling 1 set (table S8, Figure S2), random sampling 2 set (table S8, Figure S3), and random sampling 3 set (table S10, Figure S4).

### Calibration plot analysis

**Fig. 5:**
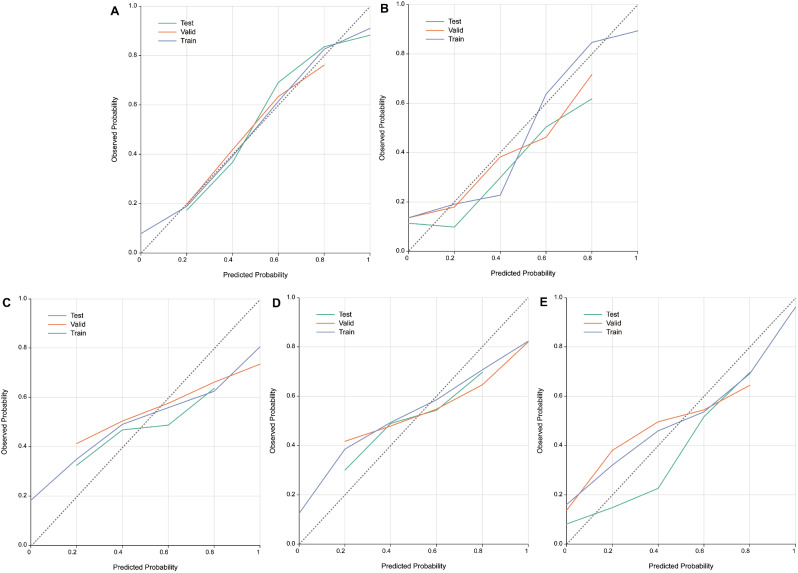
Calibration curve of GBM model. **A**: GBM model calibration based on all the variables in the training set, internal validation set and the external test set. **B**: GBM model calibration based on the nine selected variables in the training set, internal validation set and the external test set. **C**: GBM model calibration in random sample set 1 based on the nine selected variables in the training set, internal validation set and the external test set. **D**: GBM model calibration in random sample set 2 based on the nine selected variables in the training set, internal validation set and the external test set. **E**: GBM model calibration in random sample set 3 based on the nine selected variables in the training set, internal validation set and the external test set

Calibration plot analysis shows that the GBM diagnostic model had good calibration in the train set (Fig. 5A), internal validation set (Fig. 5B), external test set (Fig. 5C), and random sampling set (Set 1: Fig. 5D; Set 2: Fig. 5E; Set 3: Fig. 5F).

### The relationship between these nine features and RD

We further conducted Spearman analysis and Logistic regression analysis to explore the relationship between these nine features and RD. As shown in Table S11, older age, GLB, GLU, MPV, and UA were positively significantly associated with RD (*P* < 0.001). Conversely, male, PCT, BASP, and APTT were negatively significantly associated with RD (*P* < 0.001). Furthermore, Logistic regression analysis also showed that older age, increased levels of GLB, GLU, MPV, and UA were risk factors for RD; male gender and decreased levels of PCT, BASP, and APTT were also risk factors for RD (Table S12).

### Web server of the model

**Fig. 6:**
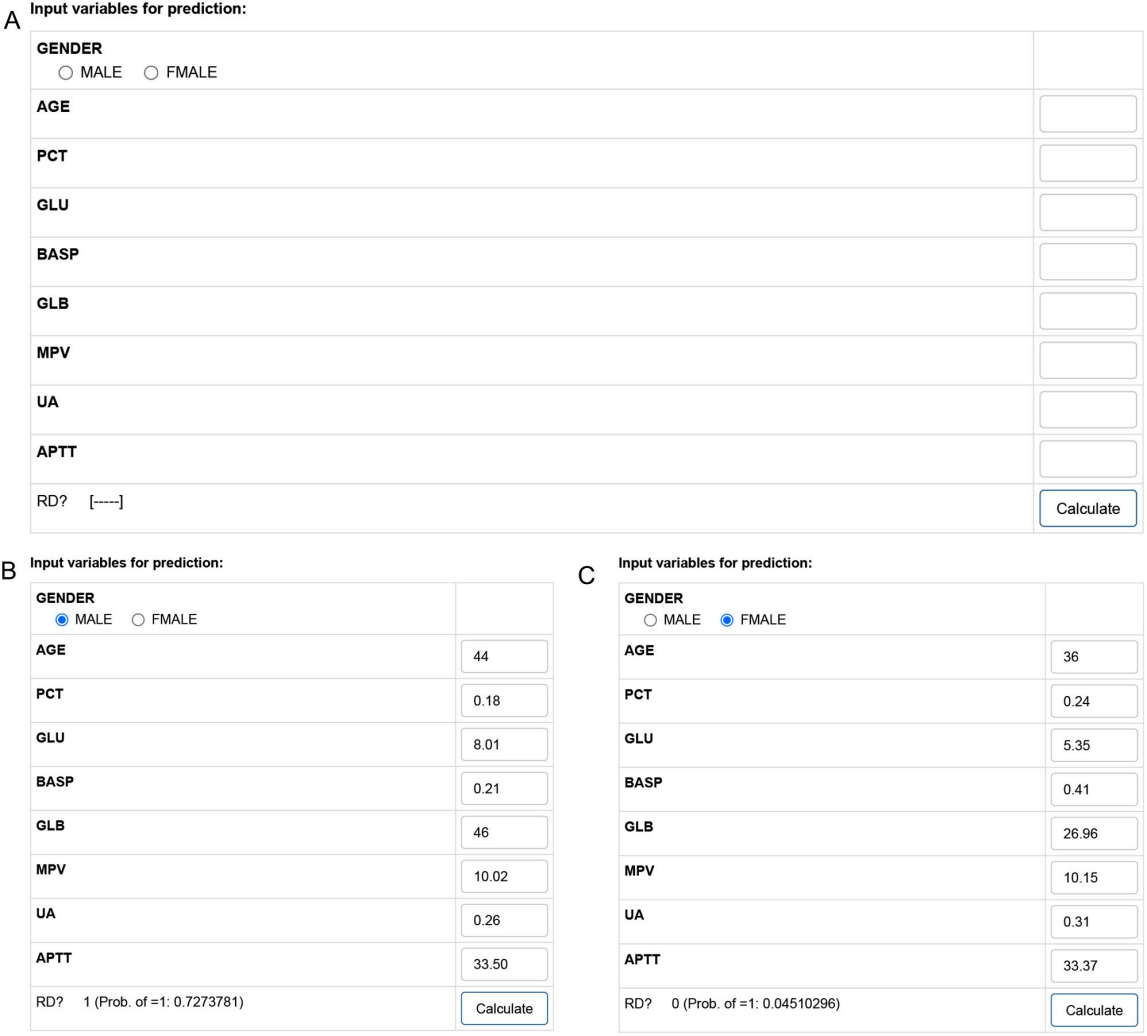
The public internet calculator for RD discrimination by nine features. The application web server of GBM model with nine features available at http://www.empowerstats.net/pmodel/?m=31141_GBM9 for the RD prediction in patients with HM (**A**). Users could predict RD by submitting nine features into the text boxes. An example of a 44-year-old male participant with PCT of 0.18, GLU of 8.01, BASP of 0.21, GLB of 46, MPV of 10.02, UA of 0.26, and APTT of 33.50, who was enrolled in the Xuhui Central hospital in 2022 is demonstrated on this webpage (Fig. 6**B**). An example of a 36-year-old female participant with PCT of 0.24, GLU of 5.35, BASP of 0.41, GLB of 26.96, MPV of 10.15, UA of 0.31, and APTT of 33.37, who was enrolled in the EENT hospital in 2023 is demonstrated on this webpage (Fig. 6**C**).

To facilitate the application of the model, we implemented the GBM-RD model into a web application (Fig. 6A) that provides risk prediction for HM individuals. Visitors might predict HMRD by entering the order of nine features into the text fields on the web page. The estimated risks of RD will be displayed at the bottom of the panel.

An example of a 44-year-old male participant with PCT of 0.18, GLU of 8.01, BASP of 0.21, GLB of 46, MPV of 10.02, UA of 0.26, and APTT of 33.50, who was enrolled in the Xuhui Central hospital in 2022 is demonstrated on this webpage (Fig. 6B). The calculated risk probability of HMRD was 0.727. An example of a 36-year-old female participant with PCT of 0.24, GLU of 5.35, BASP of 0.41, GLB of 26.96, MPV of 10.15, UA of 0.31, and APTT of 33.37, who was enrolled in the EENT hospital in 2023 is demonstrated on this webpage (Fig. 6C). The calculated risk probability of HMRD was 0.045. The web application was made accessible online athttp://www.empowerstats.net/pmodel/?m=31141_GBM9

## Discussion

HM patients seldom visit an ophthalmologist unless their symptoms increase or their eyesight abruptly deteriorates in China. As a result, it is challenging to identify and diagnose HMRD early when utilizing models based on ocular exams. So, there is still a clinical need to develop a rapid, easy, precise, and valuable screening technique to identify HMRD. To the best of our knowledge, this study is the first one that uses Clinlabomics to predict the diagnosis of RD in the HM population and has external validation.

We created a machine learning diagnostic model for RD in the HM individuals that performed well during internal and external validations. The GBM model reached an AUC of 0.8550 and an AUCPR of 0.5584 in the training set and up to 0.8405 (AUC) and 0.5355 (AUCPR) in internal validations. Interestingly, the GBM model reached an AUC of 0.7579 and an AUCPR of 0.5587 in the external test evaluation. Furthermore, the three random data sets showed that the GBM model could retain a robust performance (table s8-s10). Finally, calibration plots analysis showed that using the model in practice could provide a good agreement between predicted and observed outcomes. This study expanded our previous work using machine learning approaches to improve the diagnostic accuracy of HMRD and broaden the applicability of this model [24].

Previously, researchers have created several diagnostic models for RD and other eye diseases. Ohsugi and colleagues [10] applied a convolutional neural network algorithm to detect RD using ultra-wide-field fundus images. They achieved a high AUC of 0.988 (95% CI, 0.981–0.995) but did not include external validation. Meanwhile, their reliance on specialized eye examination tools could not be appropriate for patient screening. Recently, Nezu et al. [32] based on 28 immune mediators in aqueous humor, successfully predicted the diagnosis of RD with an AUC of 0.87 and AUCPR of 0.59. Although it showed high discrimination for RD identification, it had a minimal sample size (*n* = 52) and lack a validation set. Furthermore, there are limited opportunities to obtain aqueous humor from the health screening population. Irfahan Kassam et al. [33] reported that polygenic risk scores had an AUC of 0.66 (95% CI = 0.63–0.70) for myopic macular degeneration versus no myopia. Compared with the models established based on variables obtained from elaborate ophthalmic tests, expensive whole genome/exome sequencing, or invasive paracentesis of the anterior chamber, our model is solely based on the easily accessible diagnosis factors which can be collected from simple blood tests. Therefore, this diagnostic model can be widely applied to medical institutions at different levels.

In our diagnostic model, the nine more important variables contributing to HMRD diagnose were age, gender, APTT, BASP, GLB, GLU, MPV, PCT, and UA. In almost all models, age was consistently identified as the essential factor associated with RD diagnosis. Previous observational studies have shown that older age was an independent risk factor for the presentation of RD [34, 35]. Gender was another contributing variable to the model. Previous studies have shown that male gender should be considered an individual risk factor for RD [4, 35]. Other contributing factors included modifiable factors, such as APTT, BASP, GLB, GLU, MPV, PCT, and UA. Arndt C and colleague [22] found that intravitreal glucose concentration was higher in the RD group. Moreover, APTT, BASP, GLB, MPV, PCT, and UA indicate the whole body’s general homeostasis and inflammatory state.

The pathophysiology of RD is thought to involve several pathogenic processes, although mounting research indicates that inflammation is a crucial factor [21, 36, 37]. For example, Lin et al. [38] provide clinical and experimental evidence that inflammation plays a crucial role in the development of myopia. Transforming growth factor-β and matrix metalloproteinase 2 expression is upregulated in myopic eyes, but collagen I expression is downregulated [39].

Qin et al. [21] hypothesis that immunological/inflammatory markers, namely hs-CRP, C3, and CH50 may play an important role in the development of Pathological Myopia, and that C3 level may be a predictive risk factor for myopic choroidal neovascularization formation. A 26-year follow-up of patients with juvenile chronic arthritis found a higher percentage of these patients had myopic refractive errors than age-matched control individuals, pointing to a link between myopia and juvenile chronic arthritis [40]. The study also hypothesized that the increased prevalence of myopia was brought on by chronic inflammation, which weakened the scleral connective tissue [41]. Thus, we hypothesized that chronic systemic inflammation plays a crucial role in the development of HM and RD. Consequently, Clinlabomics data could be a novel strategy for the early identification of a high risk of RD in patients with HM.

Our study has several strengths. First, our top-performing GBM provides many benefits, including automated handling of missing data, more flexibility in hyperparameter adjustment to account for intricate interactions between predictors and outcomes, and frequently improved performance compared to other methods [42]. Second, we examined China’s most significant patient cohorts, with a total sample size of 30,047 patients. This ensures the accuracy of the statistics and reflects actual usage in our nation. Third, we applied random resampling techniques to confirm the internal validity of the results. Fourth, the Clinlabomics -based GBM model is advantageous due to its nine well-performed indexes, low cost, and clinical applicability in primary care, making it suitable for deciding who should receive detailed ophthalmic examinations for RD in patients with HM. Ultimately, integrating machine learning technology with other innovations, like the Internet of Things (IoT), offers a promising avenue to substantially improve the efficiency and reduce the costs associated with diagnosing RD. An illustrative example is the early stages of RD, during which specific blood markers may exhibit changes. This scenario allows for the detection and quantification of these markers in the bloodstream. Subsequently, the data can be transmitted to medical centers equipped with machine learning technology via IoT devices. This method enables patients to undergo diagnostic tests for RD promptly, affordably, and with minimal effort during its early stages. Consequently, individuals with concerning results can seek medical consultation swiftly.

Our study has limitations. First, during the external test, we observed a reduction in model performance by training our model on the largest (EENT) cohort and separately assessing its performance in the Xuhui Central hospital cohorts. This reduction might be due to the smaller sample size in the external test cohort [43], or it might be due to differences in the distribution of input variables and differences in the detecting instrument of input variables. Second, a class-imbalance data set with a limited number of observed occurrences (5607 of 30,047 patients) may be another study drawback, although the random sampling approach was utilized to balance medical data. Third, our GBM model only takes age, gender, and routine blood parameters as input data without incorporating ophthalmic or other clinical parameters. This is because routine blood parameters are highly feasible and widely accessible, thus allowing for integrated analysis of multiple modalities for clinical RD evaluation and diagnosis. In our study, it was observed that the HMRD patients exhibited a higher likelihood of being older (*P* < 0.05) compared to the HM patients, suggesting the potential influence of age on other factors. To investigate this further, we conducted a LASSO analysis, which revealed no significant collinearity between age and other variables (variance inflation factor < 10). Additionally, no significant associations were found between age and gender, activated partial thromboplastin time, basophil%, globulin, glucose, mean platelet volume, thrombocytosis, and uric acid (*P* > 0.05). These findings indicate that age does not impact other factors under consideration.

## Conclusion

We demonstrated that GBM algorithms based on nine features successfully predicted the diagnosis of RD in patients with HM, which retained its performance during external validation. However, further external validation is warranted to assess model performance in other populations.

### Electronic supplementary material

Below is the link to the electronic supplementary material.

Supplementary Material 1

Supplementary Material 2

Supplementary Material 3

Supplementary Material 4

Supplementary Material 5

## Data Availability

The datasets used and/or analyzed during the current study are available from the corresponding author on reasonable request.

## Abbreviations

RD: retinal detachment
Clinlabomics: clinical laboratory omics
HM: high myopia
AUC: the area under the receiver operating characteristic curve
AUCPR: the area under the precision-recall curve
D: diopter
RF: random forest
GBM: gradient boosting machine
GLM: generalized linear model
TBA: total bile acid
TBIL: total bilirubin
TC: total cholesterol
N: neutrophil number
ALB: albumin
RBC: red blood count
HBA1C: glycosylated hemoglobin
PCT: thrombocytocrit
PDW: platelet distribution width
GLU: glucose
FIB: fibrinogen
EOSP: percentage of eosinophils
GLB: globulin
PAB: prealbumin
MPV: mean platelet volume
TT: thrombin time
UA: uric acid
BUN: blood urea nitrogen
P: phosphorus
LY: lymphocyte count
ALP: alkaline phosphatase
K: kalium
CK: creatine kinase
CREA: creatinine
AST: glutamic oxalacetic transaminase
ALT: glutamic-pyruvic transaminase
GGT: gamma-glutamyl transpeptidase
TG: triglyceride
CA: calcium
MONP: percentage of monocyte
APTT: activated partial thromboplastin time
INR: international normalized ratio
RBCSD: red blood cell distribution width-standard deviation
PTP: percentage of prothrombin time
MCV: mean corpuscular volume
MCHC: mean corpuscular hemoglobin concentration
DD: d-dimer
BASP: percentage of basophil
CL: chloridion
DBIL: direct bilirubin
