# Supplementary figures and images for: Developing and validating a clinlabomics-based machine-learning model for early detection of retinal detachment in patients with high myopia
Source: J Transl Med. 2024 Apr 30;22:405. doi: 10.1186/s12967-024-05131-9 (PMC11061938; doi:10.1186/s12967-024-05131-9)

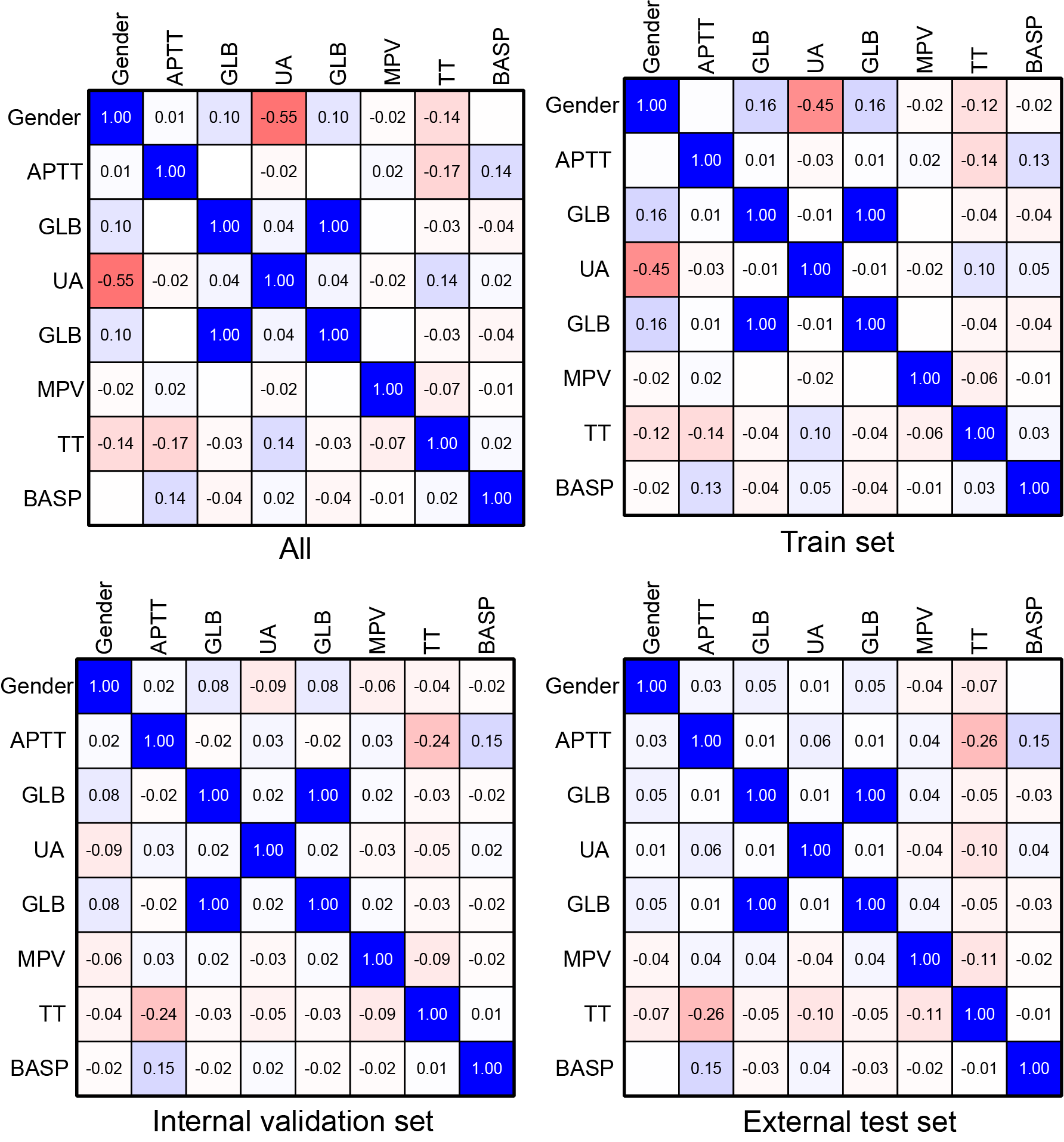

Supplement: Supplementary file 1 — Supplementary Material 1 [file 12967_2024_5131_MOESM1_ESM.tif]

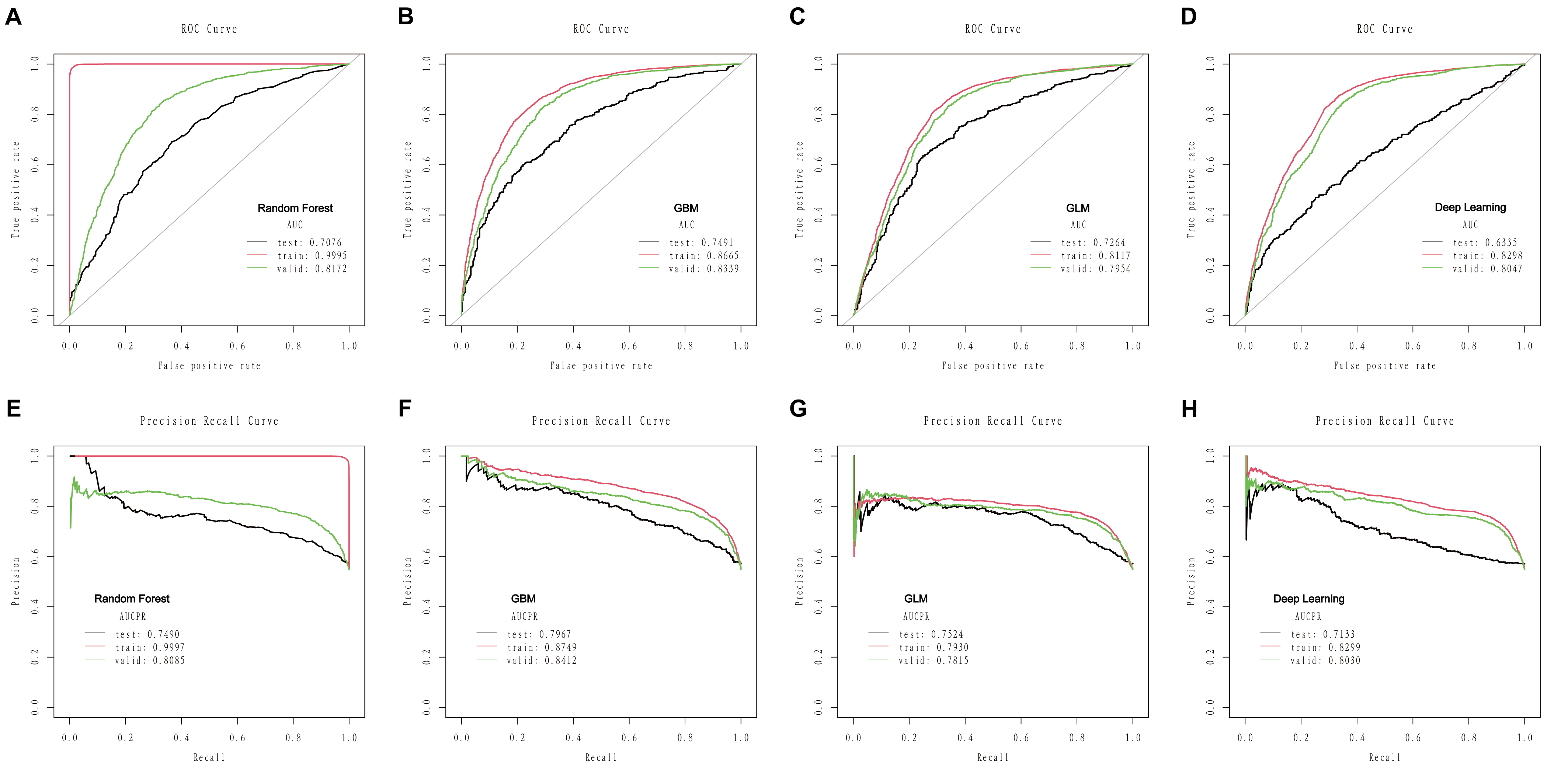

Supplement: Supplementary file 2 — Supplementary Material 2 [file 12967_2024_5131_MOESM2_ESM.tif]

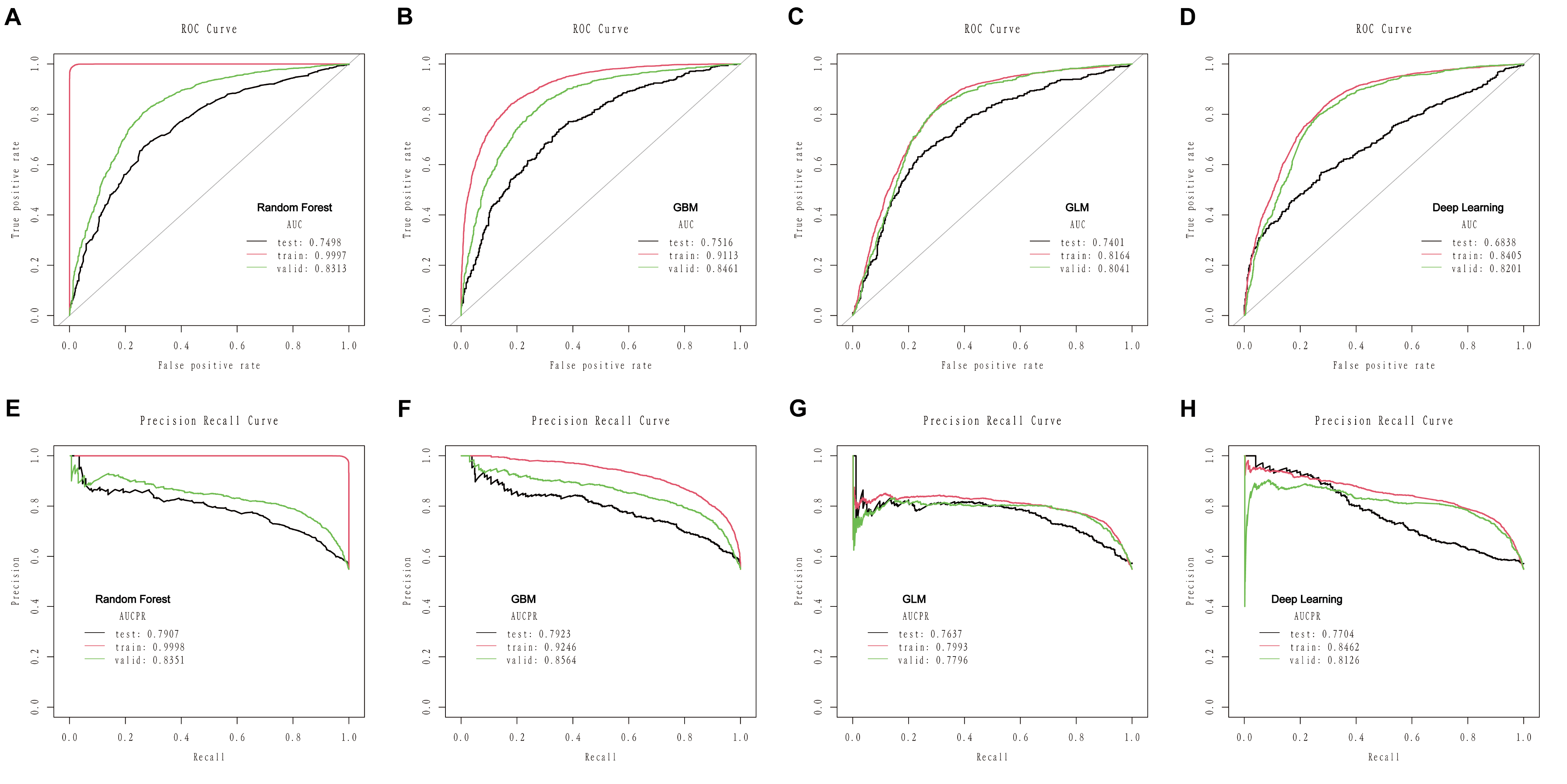

Supplement: Supplementary file 3 — Supplementary Material 3 [file 12967_2024_5131_MOESM3_ESM.tif]

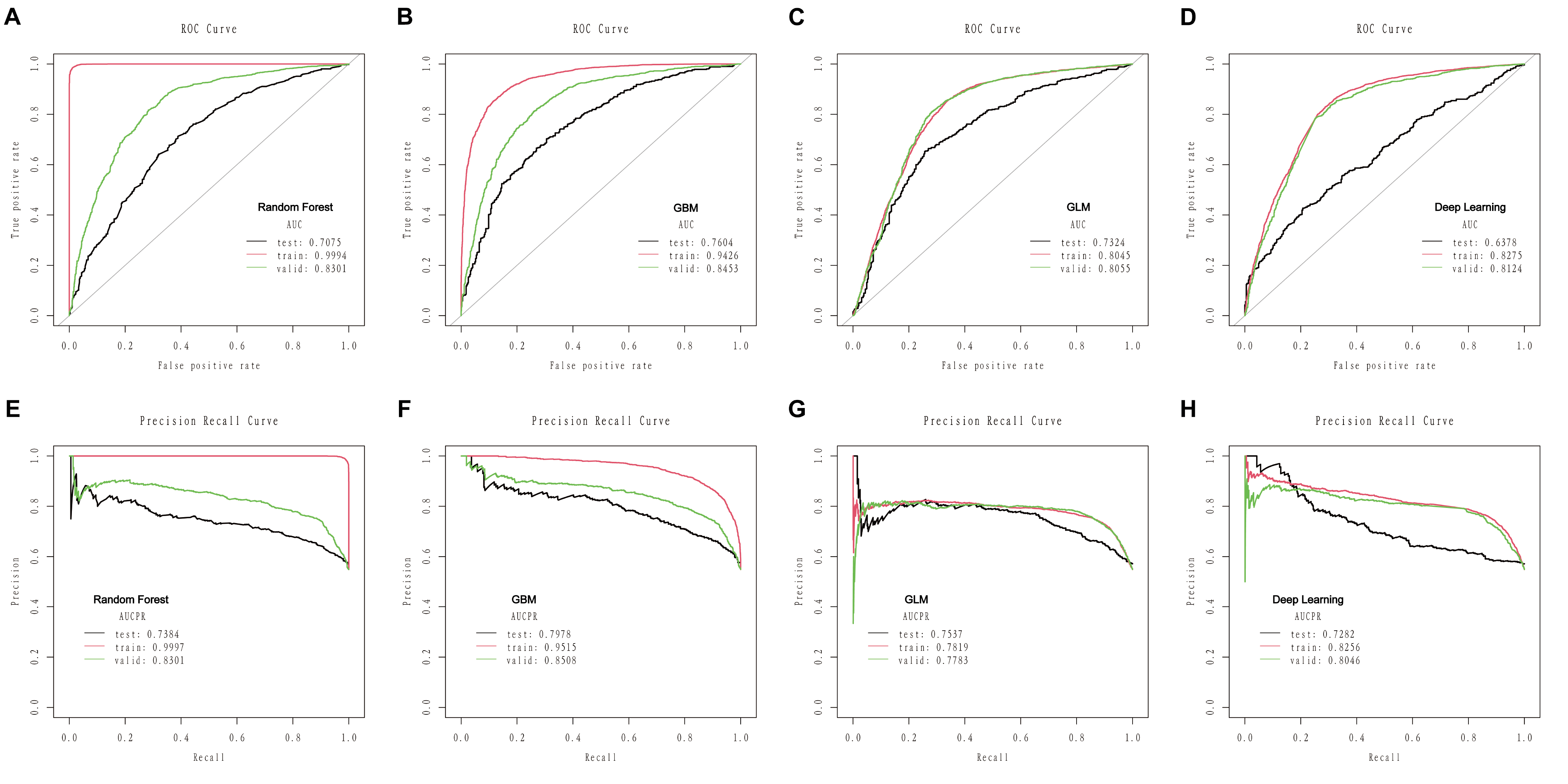

Supplement: Supplementary file 4 — Supplementary Material 4 [file 12967_2024_5131_MOESM4_ESM.tif]
