# Supplementary material for: Developing and validating a clinlabomics-based machine-learning model for early detection of retinal detachment in patients with high myopia
Source: J Transl Med. 2024 Apr 30;22:405. doi: 10.1186/s12967-024-05131-9 (PMC11061938; doi:10.1186/s12967-024-05131-9)
Supplement: Supplementary file 5 — Supplementary Material 5 [file 12967_2024_5131_MOESM5_ESM.docx]

Table S1. Baseline characteristics of all the patients

|  | Train set | Internal validation set | External test set | P-value |
| --- | --- | --- | --- | --- |
| N | 21034 | 7011 | 2002 |  |
| AGE | 39.55 ± 16.71 | 39.43 ± 16.68 | 38.61 ± 17.58 | 0.055 |
| GENDER |  |  |  | <0.001 |
| Male | 6828 (32.46%) | 2259 (32.22%) | 236 (11.79%) |  |
| Female | 14206 (67.54%) | 4752 (67.78%) | 1766 (88.21%) |  |
| TBA(μmol/L) | 3.68 ± 3.70 | 3.68 ± 3.81 | 3.85 ± 3.37 | 0.158 |
| TBIL(μmol/L) | 11.24 ± 5.40 | 11.11 ± 5.30 | 13.06 ± 5.99 | <0.001 |
| TC(mmol/L) | 4.56 ± 0.92 | 4.57 ± 0.92 | 4.77 ± 0.97 | <0.001 |
| N(10^9/L) | 3.71 ± 1.32 | 3.73 ± 1.34 | 4.12 ± 1.36 | <0.001 |
| ALB(g/L) | 48.06 ± 3.27 | 48.01 ± 3.33 | 49.20 ± 3.61 | <0.001 |
| RBC(10^12/L) | 4.54 ± 0.46 | 4.54 ± 0.46 | 5.00 ± 0.49 | <0.001 |
| HBA1C(%) | 5.42 ± 0.61 | 5.42 ± 0.62 | 5.54 ± 0.69 | <0.001 |
| PCT(%) | 0.23 ± 0.05 | 0.23 ± 0.05 | 0.24 ± 0.05 | 0.002 |
| PDW(%) | 12.44 ± 2.78 | 12.44 ± 2.77 | 12.57 ± 2.74 | 0.128 |
| GLU(mmol/L) | 5.44 ± 1.22 | 5.46 ± 1.20 | 5.61 ± 1.22 | <0.001 |
| FIB(g/L) | 2.76 ± 0.61 | 2.77 ± 0.61 | 2.81 ± 0.70 | 0.001 |
| EOSP(%) | 1.78 ± 1.61 | 1.76 ± 1.65 | 2.06 ± 1.69 | <0.001 |
| BASP(%) | 0.40 ± 0.26 | 0.40 ± 0.27 | 0.40 ± 0.25 | 0.988 |
| LDH(U/L) | 156.72 ± 30.61 | 157.45 ± 31.05 | 168.10 ± 36.75 | <0.001 |
| GLB(g/L) | 27.16 ± 4.25 | 27.16 ± 4.25 | 27.58 ± 4.39 | <0.001 |
| PAB(mg/L) | 272.57 ± 56.25 | 273.43 ± 55.88 | 325.75 ± 69.21 | <0.001 |
| MPV(fL) | 10.18 ± 1.14 | 10.16 ± 1.13 | 10.15 ± 1.12 | 0.249 |
| TT(s) | 17.99 ± 1.21 | 17.98 ± 1.38 | 18.41 ± 1.16 | <0.001 |
| UA(mmol/L) | 0.30 ± 0.07 | 0.30 ± 0.07 | 0.51 ± 0.05 | <0.001 |
| BUN(mmol/L) | 4.84 ± 1.38 | 4.84 ± 1.41 | 5.45 ± 1.65 | <0.001 |
| SODIUM(mmol/L) | 141.01 ± 2.13 | 141.01 ± 2.13 | 141.52 ± 2.11 | <0.001 |
| CL(mmol/L) | 102.84 ± 2.35 | 102.85 ± 2.33 | 102.31 ± 2.58 | <0.001 |
| P(mmol/L) | 1.16 ± 0.17 | 1.17 ± 0.17 | 1.15 ± 0.18 | 0.005 |
| LY(10^9/L) | 1.87 ± 0.57 | 1.88 ± 0.63 | 2.04 ± 0.64 | <0.001 |
| ALP(U/L) | 67.16 ± 22.03 | 66.74 ± 21.05 | 75.67 ± 23.14 | <0.001 |
| K(mmol/L) | 4.22 ± 0.34 | 4.21 ± 0.35 | 4.28 ± 0.37 | <0.001 |
| CK(U/L) | 110.56 ± 281.07 | 110.70 ± 248.30 | 159.19 ± 267.42 | <0.001 |
| CREA(μmol/L) | 66.11 ± 17.95 | 66.11 ± 15.80 | 86.00 ± 21.99 | <0.001 |
| AST(U/L) | 19.19 ± 10.39 | 19.11 ± 9.27 | 25.43 ± 18.94 | <0.001 |
| ALT(U/L) | 18.82 ± 18.24 | 18.59 ± 17.86 | 36.37 ± 31.66 | <0.001 |
| GGT(U/L) | 21.90 ± 24.73 | 21.70 ± 24.18 | 42.34 ± 48.55 | <0.001 |
| TG(mmol/L) | 1.27 ± 0.99 | 1.28 ± 1.07 | 2.05 ± 1.62 | <0.001 |
| CA(mmol/L) | 2.35 ± 0.10 | 2.35 ± 0.10 | 2.40 ± 0.11 | <0.001 |
| MONP(%) | 6.44 ± 1.73 | 6.39 ± 1.69 | 6.80 ± 1.72 | <0.001 |
| APTT(s) | 33.41 ± 5.63 | 33.33 ± 5.75 | 32.74 ± 6.00 | <0.001 |
| INR | 0.97 ± 0.08 | 0.96 ± 0.08 | 0.96 ± 0.08 | <0.001 |
| RBCSD(fL) | 40.77 ± 2.93 | 40.74 ± 2.94 | 40.13 ± 2.96 | <0.001 |
| PTP(%) | 107.04 ± 12.72 | 107.33 ± 12.98 | 108.78 ± 13.43 | <0.001 |
| MCV(fL) | 89.40 ± 4.90 | 89.40 ± 4.92 | 88.73 ± 4.48 | <0.001 |
| MCHC(g/L) | 334.70 ± 11.61 | 334.62 ± 11.57 | 339.75 ± 11.11 | <0.001 |
| DD(μg/mL) | 2.75 ± 39.05 | 3.68 ± 55.71 | 3.89 ± 55.55 | 0.217 |
| DBIL(μmol/L) | 4.38 ± 1.82 | 4.33 ± 1.80 | 4.95 ± 1.97 | <0.001 |

TBA: total bile acid; TBIL: total bilirubin; TC: total cholesterol; N: neutrophil number; ALB: albumin; RBC: red blood count; HBA1C: glycosylated hemoglobin; PCT: thrombocytocrit; PDW: platelet distribution width; GLU: glucose; FIB: fibrinogen; EOSP: percentage of eosinophils; GLB: globulin; PAB: prealbumin; MPV: mean platelet volume; TT: thrombin time; UA: uric acid; BUN: blood urea nitrogen; P: phosphorus; LY: lymphocyte count; ALP: alkaline phosphatase; K: kalium; CK: creatine kinase; CREA: creatinine; AST: glutamic oxalacetic transaminase; ALT: glutamic-pyruvic transaminase; GGT: gamma-glutamyl transpeptidase; TG: triglyceride; CA: calcium; MONP: percentage of monocyte; APTT: activated partial thromboplastin time; INR: international normalized ratio; RBCSD: red blood cell distribution width-standard deviation; PTP: percentage of prothrombin time; MCV: mean corpuscular volume; MCHC: mean corpuscular hemoglobin concentration; DD: d-dimer; BASP: percentage of basophil; CL: chloridion; DBIL: direct bilirubin.

Table S2. Baseline characteristics of HM patients

|  | Train set | Internal validation set | External test set | P-value |
| --- | --- | --- | --- | --- |
| N | 17259 | 5694 | 1487 |  |
| AGE | 36.80 ± 15.95 | 36.64 ± 15.96 | 35.60 ± 16.69 | 0.020 |
| GENDER |  |  |  | <0.001 |
| Male | 4799 (27.81%) | 1571 (27.59%) | 185 (12.44%) |  |
| Female | 12460 (72.19%) | 4123 (72.41%) | 1302 (87.56%) |  |
| TBA(μmol/L) | 3.56 ± 3.54 | 3.50 ± 3.63 | 3.71 ± 3.30 | 0.107 |
| TBIL(μmol/L) | 11.12 ± 5.34 | 11.02 ± 5.28 | 13.10 ± 5.97 | <0.001 |
| TC(mmol/L) | 4.52 ± 0.90 | 4.53 ± 0.90 | 4.74 ± 0.95 | <0.001 |
| N(10^9/L) | 3.66 ± 1.31 | 3.68 ± 1.32 | 4.07 ± 1.36 | <0.001 |
| ALB(g/L) | 48.20 ± 3.26 | 48.15 ± 3.35 | 49.48 ± 3.58 | <0.001 |
| RBC(10^12/L) | 4.52 ± 0.46 | 4.52 ± 0.45 | 5.03 ± 0.49 | <0.001 |
| HBA1C(%) | 5.36 ± 0.56 | 5.36 ± 0.56 | 5.50 ± 0.67 | <0.001 |
| PCT(%) | 0.23 ± 0.05 | 0.24 ± 0.05 | 0.24 ± 0.05 | 0.003 |
| PDW(%) | 12.33 ± 2.71 | 12.32 ± 2.72 | 12.53 ± 2.67 | 0.018 |
| GLU(mmol/L) | 5.34 ± 1.10 | 5.34 ± 1.07 | 5.54 ± 1.17 | <0.001 |
| FIB(g/L) | 2.73 ± 0.58 | 2.74 ± 0.59 | 2.74 ± 0.65 | 0.341 |
| EOSP(%) | 1.80 ± 1.60 | 1.77 ± 1.62 | 2.10 ± 1.74 | <0.001 |
| BASP(%) | 0.41 ± 0.26 | 0.41 ± 0.27 | 0.41 ± 0.25 | 0.944 |
| LDH(U/L) | 154.66 ± 29.83 | 155.38 ± 30.30 | 167.24 ± 38.50 | <0.001 |
| GLB(g/L) | 26.94 ± 4.19 | 26.94 ± 4.19 | 27.23 ± 4.31 | 0.032 |
| PAB(mg/L) | 268.07 ± 52.85 | 268.23 ± 52.03 | 322.97 ± 64.55 | <0.001 |
| MPV(fL) | 10.16 ± 1.12 | 10.15 ± 1.12 | 10.13 ± 1.09 | 0.465 |
| TT(s) | 17.99 ± 1.16 | 17.99 ± 1.43 | 18.51 ± 1.16 | <0.001 |
| UA(mmol/L) | 0.30 ± 0.07 | 0.30 ± 0.07 | 0.51 ± 0.05 | <0.001 |
| BUN(mmol/L) | 4.71 ± 1.32 | 4.71 ± 1.31 | 5.32 ± 1.55 | <0.001 |
| SODIUM(mmol/L) | 140.87 ± 2.06 | 140.87 ± 2.05 | 141.40 ± 2.04 | <0.001 |
| CL(mmol/L) | 102.83 ± 2.31 | 102.85 ± 2.28 | 102.20 ± 2.49 | <0.001 |
| P(mmol/L) | 1.17 ± 0.17 | 1.18 ± 0.17 | 1.16 ± 0.18 | <0.001 |
| LY(10^9/L) | 1.88 ± 0.56 | 1.89 ± 0.56 | 2.07 ± 0.64 | <0.001 |
| ALP(U/L) | 64.94 ± 20.90 | 64.56 ± 20.18 | 75.02 ± 23.95 | <0.001 |
| K(mmol/L) | 4.22 ± 0.33 | 4.22 ± 0.34 | 4.28 ± 0.36 | <0.001 |
| CK(U/L) | 112.66 ± 308.27 | 113.31 ± 273.28 | 172.70 ± 306.10 | <0.001 |
| CREA(μmol/L) | 65.20 ± 15.76 | 65.10 ± 14.77 | 84.82 ± 18.57 | <0.001 |
| AST(U/L) | 18.78 ± 10.32 | 18.77 ± 9.28 | 25.48 ± 20.52 | <0.001 |
| ALT(U/L) | 17.88 ± 17.80 | 17.69 ± 17.05 | 36.57 ± 31.66 | <0.001 |
| GGT(U/L) | 20.28 ± 21.73 | 20.31 ± 23.67 | 41.97 ± 51.58 | <0.001 |
| TG(mmol/L) | 1.18 ± 0.92 | 1.19 ± 1.01 | 1.95 ± 1.47 | <0.001 |
| CA(mmol/L) | 2.35 ± 0.10 | 2.35 ± 0.10 | 2.41 ± 0.11 | <0.001 |
| MONP(%) | 6.45 ± 1.72 | 6.40 ± 1.68 | 6.84 ± 1.70 | <0.001 |
| APTT(s) | 33.46 ± 5.67 | 33.35 ± 5.71 | 32.43 ± 6.09 | <0.001 |
| INR | 0.97 ± 0.08 | 0.97 ± 0.08 | 0.95 ± 0.07 | <0.001 |
| RBCSD(fL) | 40.63 ± 2.87 | 40.58 ± 2.88 | 39.86 ± 2.81 | <0.001 |
| PTP(%) | 106.67 ± 12.58 | 106.97 ± 12.81 | 108.75 ± 13.46 | <0.001 |
| MCV(fL) | 89.26 ± 4.89 | 89.24 ± 4.87 | 88.44 ± 4.50 | <0.001 |
| MCHC(g/L) | 334.27 ± 11.50 | 334.27 ± 11.59 | 339.45 ± 11.29 | <0.001 |
| DD(μg/mL) | 0.50 ± 9.04 | 0.32 ± 2.28 | 0.48 ± 8.68 | 0.329 |
| DBIL(μmol/L) | 4.40 ± 1.81 | 4.37 ± 1.79 | 5.03 ± 1.94 | <0.001 |

TBA: total bile acid; TBIL: total bilirubin; TC: total cholesterol; N: neutrophil number; ALB: albumin; RBC: red blood count; HBA1C: glycosylated hemoglobin; PCT: thrombocytocrit; PDW: platelet distribution width; GLU: glucose; FIB: fibrinogen; EOSP: percentage of eosinophils; GLB: globulin; PAB: prealbumin; MPV: mean platelet volume; TT: thrombin time; UA: uric acid; BUN: blood urea nitrogen; P: phosphorus; LY: lymphocyte count; ALP: alkaline phosphatase; K: kalium; CK: creatine kinase; CREA: creatinine; AST: glutamic oxalacetic transaminase; ALT: glutamic-pyruvic transaminase; GGT: gamma-glutamyl transpeptidase; TG: triglyceride; CA: calcium; MONP: percentage of monocyte; APTT: activated partial thromboplastin time; INR: international normalized ratio; RBCSD: red blood cell distribution width-standard deviation; PTP: percentage of prothrombin time; MCV: mean corpuscular volume; MCHC: mean corpuscular hemoglobin concentration; DD: d-dimer; BASP: percentage of basophil; CL: chloridion; DBIL: direct bilirubin

Table S3. Baseline characteristics of HMRD patients

|  | Train set | Internal validation set | External test set | P-value |
| --- | --- | --- | --- | --- |
| N | 3775 | 1317 | 515 |  |
| AGE | 52.13 ± 14.15 | 51.50 ± 14.17 | 47.31 ± 17.20 | <0.001 |
| GENDER |  |  |  | <0.001 |
| Male | 2029 (53.75%) | 688 (52.24%) | 51 (9.90%) |  |
| Female | 1746 (46.25%) | 629 (47.76%) | 464 (90.10%) |  |
| TBA(μmol/L) | 4.25 ± 4.33 | 4.48 ± 4.41 | 4.23 ± 3.54 | 0.221 |
| TBIL(μmol/L) | 11.78 ± 5.64 | 11.52 ± 5.36 | 12.95 ± 6.03 | <0.001 |
| TC(mmol/L) | 4.75 ± 0.97 | 4.78 ± 1.01 | 4.86 ± 1.01 | 0.064 |
| N(10^9/L) | 3.94 ± 1.35 | 3.95 ± 1.39 | 4.26 ± 1.33 | <0.001 |
| ALB(g/L) | 47.43 ± 3.23 | 47.40 ± 3.21 | 48.36 ± 3.58 | <0.001 |
| RBC(10^12/L) | 4.64 ± 0.47 | 4.64 ± 0.48 | 4.91 ± 0.48 | <0.001 |
| HBA1C(%) | 5.65 ± 0.76 | 5.64 ± 0.79 | 5.64 ± 0.73 | 0.891 |
| PCT(%) | 0.22 ± 0.05 | 0.22 ± 0.05 | 0.23 ± 0.05 | 0.005 |
| PDW(%) | 12.96 ± 3.01 | 12.96 ± 2.92 | 12.69 ± 2.94 | 0.142 |
| GLU(mmol/L) | 5.94 ± 1.57 | 6.00 ± 1.53 | 5.82 ± 1.35 | 0.066 |
| FIB(g/L) | 2.93 ± 0.67 | 2.92 ± 0.71 | 3.02 ± 0.78 | 0.017 |
| EOSP(%) | 1.69 ± 1.63 | 1.74 ± 1.76 | 1.93 ± 1.54 | 0.008 |
| BASP(%) | 0.34 ± 0.27 | 0.35 ± 0.29 | 0.37 ± 0.26 | 0.076 |
| LDH(U/L) | 166.13 ± 32.32 | 166.38 ± 32.63 | 170.59 ± 31.07 | 0.013 |
| GLB(g/L) | 28.14 ± 4.36 | 28.11 ± 4.37 | 28.58 ± 4.49 | 0.083 |
| PAB(mg/L) | 293.14 ± 65.90 | 295.92 ± 65.57 | 333.79 ± 80.71 | <0.001 |
| MPV(fL) | 10.26 ± 1.20 | 10.20 ± 1.16 | 10.21 ± 1.20 | 0.202 |
| TT(s) | 17.97 ± 1.43 | 17.94 ± 1.14 | 18.11 ± 1.13 | 0.048 |
| UA(mmol/L) | 0.31 ± 0.07 | 0.31 ± 0.07 | 0.50 ± 0.05 | <0.001 |
| BUN(mmol/L) | 5.42 ± 1.52 | 5.39 ± 1.65 | 5.83 ± 1.88 | <0.001 |
| SODIUM(mmol/L) | 141.66 ± 2.31 | 141.65 ± 2.34 | 141.87 ± 2.28 | 0.141 |
| CL(mmol/L) | 102.90 ± 2.56 | 102.87 ± 2.51 | 102.62 ± 2.81 | 0.065 |
| P(mmol/L) | 1.13 ± 0.17 | 1.12 ± 0.18 | 1.14 ± 0.18 | 0.385 |
| LY(10^9/L) | 1.82 ± 0.62 | 1.81 ± 0.87 | 1.97 ± 0.64 | <0.001 |
| ALP(U/L) | 77.32 ± 24.14 | 76.16 ± 22.13 | 77.56 ± 20.52 | 0.266 |
| K(mmol/L) | 4.21 ± 0.37 | 4.21 ± 0.38 | 4.28 ± 0.39 | <0.001 |
| CK(U/L) | 100.95 ± 74.91 | 99.42 ± 71.96 | 120.19 ± 73.96 | <0.001 |
| CREA(μmol/L) | 70.27 ± 25.29 | 70.48 ± 19.04 | 89.42 ± 29.48 | <0.001 |
| AST(U/L) | 21.07 ± 10.49 | 20.59 ± 9.08 | 25.29 ± 13.34 | <0.001 |
| ALT(U/L) | 23.11 ± 19.61 | 22.49 ± 20.58 | 35.76 ± 31.69 | <0.001 |
| GGT(U/L) | 29.29 ± 34.38 | 27.70 ± 25.42 | 43.40 ± 38.54 | <0.001 |
| TG(mmol/L) | 1.65 ± 1.21 | 1.68 ± 1.23 | 2.35 ± 1.96 | <0.001 |
| CA(mmol/L) | 2.35 ± 0.10 | 2.35 ± 0.10 | 2.39 ± 0.12 | <0.001 |
| MONP(%) | 6.40 ± 1.78 | 6.33 ± 1.71 | 6.67 ± 1.77 | <0.001 |
| APTT(s) | 33.19 ± 5.45 | 33.24 ± 5.92 | 33.63 ± 5.64 | 0.241 |
| INR | 0.96 ± 0.09 | 0.96 ± 0.07 | 0.96 ± 0.10 | 0.834 |
| RBCSD(fL) | 41.38 ± 3.12 | 41.42 ± 3.08 | 40.93 ± 3.22 | 0.006 |
| PTP(%) | 108.71 ± 13.21 | 108.88 ± 13.58 | 108.87 ± 13.38 | 0.912 |
| MCV(fL) | 90.05 ± 4.93 | 90.11 ± 5.05 | 89.56 ± 4.30 | 0.083 |
| MCHC(g/L) | 336.67 ± 11.90 | 336.16 ± 11.38 | 340.63 ± 10.52 | <0.001 |
| DD(μg/mL) | 13.02 ± 89.43 | 18.19 ± 127.48 | 13.74 ± 107.99 | 0.278 |
| DBIL(μmol/L) | 4.31 ± 1.86 | 4.18 ± 1.84 | 4.70 ± 2.03 | <0.001 |

TBA: total bile acid; TBIL: total bilirubin; TC: total cholesterol; N: neutrophil number; ALB: albumin; RBC: red blood count; HBA1C: glycosylated hemoglobin; PCT: thrombocytocrit; PDW: platelet distribution width; GLU: glucose; FIB: fibrinogen; EOSP: percentage of eosinophils; GLB: globulin; PAB: prealbumin; MPV: mean platelet volume; TT: thrombin time; UA: uric acid; BUN: blood urea nitrogen; P: phosphorus; LY: lymphocyte count; ALP: alkaline phosphatase; K: kalium; CK: creatine kinase; CREA: creatinine; AST: glutamic oxalacetic transaminase; ALT: glutamic-pyruvic transaminase; GGT: gamma-glutamyl transpeptidase; TG: triglyceride; CA: calcium; MONP: percentage of monocyte; APTT: activated partial thromboplastin time; INR: international normalized ratio; RBCSD: red blood cell distribution width-standard deviation; PTP: percentage of prothrombin time; MCV: mean corpuscular volume; MCHC: mean corpuscular hemoglobin concentration; DD: d-dimer; BASP: percentage of basophil; CL: chloridion; DBIL: direct bilirubin

Table S4. Compare baseline characteristics between HM and HMRD group in all the subjects

|  | HM | HMRD | P-value |
| --- | --- | --- | --- |
| N | 24440 | 5607 |  |
| AGE | 36.69 ± 16.00 | 51.54 ± 14.52 | <0.001 |
| GENDER |  |  | <0.001 |
| Male | 7672 (31.39%) | 3181 (56.73%) |  |
| Female | 16768 (68.61%) | 2426 (43.27%) |  |
| TBA(μmol/L) | 3.55 ± 3.55 | 4.30 ± 4.28 | <0.001 |
| TBIL(μmol/L) | 11.22 ± 5.39 | 11.83 ± 5.62 | <0.001 |
| TC(mmol/L) | 4.53 ± 0.90 | 4.77 ± 0.98 | <0.001 |
| N(10^9/L) | 3.69 ± 1.32 | 3.97 ± 1.36 | <0.001 |
| ALB(g/L) | 48.26 ± 3.32 | 47.51 ± 3.27 | <0.001 |
| RBC(10^12/L) | 4.55 ± 0.47 | 4.67 ± 0.48 | <0.001 |
| HBA1C(%) | 5.37 ± 0.57 | 5.65 ± 0.77 | <0.001 |
| PCT(%) | 0.24 ± 0.05 | 0.22 ± 0.05 | <0.001 |
| PDW(%) | 12.34 ± 2.71 | 12.93 ± 2.98 | <0.001 |
| GLU(mmol/L) | 5.35 ± 1.10 | 5.94 ± 1.54 | <0.001 |
| FIB(g/L) | 2.73 ± 0.59 | 2.93 ± 0.69 | <0.001 |
| EOSP(%) | 1.81 ± 1.62 | 1.72 ± 1.66 | <0.001 |
| BASP(%) | 0.41 ± 0.26 | 0.34 ± 0.27 | <0.001 |
| LDH(U/L) | 155.60 ± 30.68 | 166.60 ± 32.30 | <0.001 |
| GLB(g/L) | 26.96 ± 4.20 | 28.18 ± 4.38 | <0.001 |
| PAB(mg/L) | 271.45 ± 55.03 | 297.53 ± 68.30 | <0.001 |
| MPV(fL) | 10.15 ± 1.12 | 10.24 ± 1.19 | <0.001 |
| TT(s) | 18.02 ± 1.23 | 17.98 ± 1.35 | 0.028 |
| UA(mmol/L) | 0.31 ± 0.09 | 0.33 ± 0.09 | <0.001 |
| BUN(mmol/L) | 4.75 ± 1.34 | 5.45 ± 1.59 | <0.001 |
| SODIUM(mmol/L) | 140.90 ± 2.06 | 141.68 ± 2.32 | <0.001 |
| CL(mmol/L) | 102.80 ± 2.32 | 102.87 ± 2.57 | 0.039 |
| P(mmol/L) | 1.17 ± 0.17 | 1.13 ± 0.18 | <0.001 |
| LY(10^9/L) | 1.90 ± 0.57 | 1.83 ± 0.69 | <0.001 |
| ALP(U/L) | 65.46 ± 21.07 | 77.07 ± 23.37 | <0.001 |
| K(mmol/L) | 4.22 ± 0.34 | 4.22 ± 0.38 | 0.364 |
| CK(U/L) | 116.46 ± 300.68 | 102.36 ± 74.35 | <0.001 |
| CREA(μmol/L) | 66.37 ± 16.41 | 72.08 ± 25.02 | <0.001 |
| AST(U/L) | 19.18 ± 11.11 | 21.34 ± 10.56 | <0.001 |
| ALT(U/L) | 18.97 ± 19.30 | 24.13 ± 21.54 | <0.001 |
| GGT(U/L) | 21.61 ± 25.54 | 30.21 ± 33.19 | <0.001 |
| TG(mmol/L) | 1.23 ± 1.00 | 1.72 ± 1.32 | <0.001 |
| CA(mmol/L) | 2.36 ± 0.10 | 2.36 ± 0.11 | 0.339 |
| MONP(%) | 6.46 ± 1.72 | 6.41 ± 1.76 | 0.048 |
| APTT(s) | 33.37 ± 5.71 | 33.24 ± 5.58 | 0.121 |
| INR | 0.97 ± 0.08 | 0.96 ± 0.08 | 0.019 |
| RBCSD(fL) | 40.57 ± 2.88 | 41.35 ± 3.12 | <0.001 |
| PTP(%) | 106.87 ± 12.70 | 108.76 ± 13.31 | <0.001 |
| MCV(fL) | 89.21 ± 4.86 | 90.02 ± 4.90 | <0.001 |
| MCHC(g/L) | 334.58 ± 11.57 | 336.92 ± 11.72 | <0.001 |
| DD(μg/mL) | 0.46 ± 7.97 | 14.30 ± 101.36 | <0.001 |
| DBIL(μmol/L) | 4.43 ± 1.82 | 4.31 ± 1.88 | <0.001 |

TBA: total bile acid; TBIL: total bilirubin; TC: total cholesterol; N: neutrophil number; ALB: albumin; RBC: red blood count; HBA1C: glycosylated hemoglobin; PCT: thrombocytocrit; PDW: platelet distribution width; GLU: glucose; FIB: fibrinogen; EOSP: percentage of eosinophils; GLB: globulin; PAB: prealbumin; MPV: mean platelet volume; TT: thrombin time; UA: uric acid; BUN: blood urea nitrogen; P: phosphorus; LY: lymphocyte count; ALP: alkaline phosphatase; K: kalium; CK: creatine kinase; CREA: creatinine; AST: glutamic oxalacetic transaminase; ALT: glutamic-pyruvic transaminase; GGT: gamma-glutamyl transpeptidase; TG: triglyceride; CA: calcium; MONP: percentage of monocyte; APTT: activated partial thromboplastin time; INR: international normalized ratio; RBCSD: red blood cell distribution width-standard deviation; PTP: percentage of prothrombin time; MCV: mean corpuscular volume; MCHC: mean corpuscular hemoglobin concentration; DD: d-dimer; BASP: percentage of basophil; CL: chloridion; DBIL: direct bilirubin

Table S5. Compare baseline characteristics between HM and HMRD group in train set

|  | HM | HMRD | P-value |
| --- | --- | --- | --- |
| N | 17259 | 3775 |  |
| AGE | 36.80 ± 15.95 | 52.13 ± 14.15 | <0.001 |
| GENDER |  |  | <0.001 |
| Male | 4799 (27.81%) | 2029 (53.75%) |  |
| Female | 12460 (72.19%) | 1746 (46.25% |  |
| TBA(μmol/L) | 3.56 ± 3.54 | 4.25 ± 4.33 | <0.001 |
| TBIL(μmol/L) | 11.12 ± 5.34 | 11.78 ± 5.64 | <0.001 |
| TC(mmol/L) | 4.52 ± 0.90 | 4.75 ± 0.97 | <0.001 |
| N(10^9/L) | 3.66 ± 1.31 | 3.94 ± 1.35 | <0.001 |
| ALB(g/L) | 48.20 ± 3.26 | 47.43 ± 3.23 | <0.001 |
| RBC(10^12/L) | 4.52 ± 0.46 | 4.64 ± 0.47 | <0.001 |
| HBA1C(%) | 5.36 ± 0.56 | 5.65 ± 0.76 | <0.001 |
| PCT(%) | 0.23 ± 0.05 | 0.22 ± 0.05 | <0.001 |
| PDW(%) | 12.33 ± 2.71 | 12.96 ± 3.01 | <0.001 |
| GLU(mmol/L) | 5.34 ± 1.10 | 5.94 ± 1.57 | <0.001 |
| FIB(g/L) | 2.73 ± 0.58 | 2.93 ± 0.67 | <0.001 |
| EOSP(%) | 1.80 ± 1.60 | 1.69 ± 1.63 | <0.001 |
| BASP(%) | 0.41 ± 0.26 | 0.34 ± 0.27 | <0.001 |
| LDH(U/L) | 154.66 ± 29.83 | 166.13 ± 32.32 | <0.001 |
| GLB(g/L) | 26.94 ± 4.19 | 28.14 ± 4.36 | <0.001 |
| PAB(mg/L) | 268.07 ± 52.85 | 293.14 ± 65.90 | <0.001 |
| MPV(fL) | 10.16 ± 1.12 | 10.26 ± 1.20 | <0.001 |
| TT(s) | 17.99 ± 1.16 | 17.97 ± 1.43 | 0.525 |
| UA(mmol/L) | 0.30 ± 0.07 | 0.31 ± 0.07 | <0.001 |
| BUN(mmol/L) | 4.71 ± 1.32 | 5.42 ± 1.52 | <0.001 |
| SODIUM(mmol/L) | 140.87 ± 2.06 | 141.66 ± 2.31 | <0.001 |
| CL(mmol/L) | 102.83 ± 2.31 | 102.90 ± 2.56 | 0.087 |
| P(mmol/L) | 1.17 ± 0.17 | 1.13 ± 0.17 | <0.001 |
| LY(10^9/L) | 1.88 ± 0.56 | 1.82 ± 0.62 | <0.001 |
| ALP(U/L) | 64.94 ± 20.90 | 77.32 ± 24.14 | <0.001 |
| K(mmol/L) | 4.22 ± 0.33 | 4.21 ± 0.37 | 0.229 |
| CK(U/L) | 112.66 ± 308.27 | 100.95 ± 74.91 | 0.021 |
| CREA(μmol/L) | 65.20 ± 15.76 | 70.27 ± 25.29 | <0.001 |
| AST(U/L) | 18.78 ± 10.32 | 21.07 ± 10.49 | <0.001 |
| ALT(U/L) | 17.88 ± 17.80 | 23.11 ± 19.61 | <0.001 |
| GGT(U/L) | 20.28 ± 21.73 | 29.29 ± 34.38 | <0.001 |
| TG(mmol/L) | 1.18 ± 0.92 | 1.65 ± 1.21 | <0.001 |
| CA(mmol/L) | 2.35 ± 0.10 | 2.35 ± 0.10 | 0.491 |
| MONP(%) | 6.45 ± 1.72 | 6.40 ± 1.78 | 0.153 |
| APTT(s) | 33.46 ± 5.67 | 33.19 ± 5.45 | 0.008 |
| INR | 0.97 ± 0.08 | 0.96 ± 0.09 | 0.016 |
| RBCSD(fL) | 40.63 ± 2.87 | 41.38 ± 3.12 | <0.001 |
| PTP(%) | 106.67 ± 12.58 | 108.71 ± 13.21 | <0.001 |
| MCV(fL) | 89.26 ± 4.89 | 90.05 ± 4.93 | <0.001 |
| MCHC(g/L) | 334.27 ± 11.50 | 336.67 ± 11.90 | <0.001 |
| DD(μg/mL) | 0.50 ± 9.04 | 13.02 ± 89.43 | <0.001 |
| DBIL(μmol/L) | 4.40 ± 1.81 | 4.31 ± 1.86 | 0.004 |

TBA: total bile acid; TBIL: total bilirubin; TC: total cholesterol; N: neutrophil number; ALB: albumin; RBC: red blood count; HBA1C: glycosylated hemoglobin; PCT: thrombocytocrit; PDW: platelet distribution width; GLU: glucose; FIB: fibrinogen; EOSP: percentage of eosinophils; GLB: globulin; PAB: prealbumin; MPV: mean platelet volume; TT: thrombin time; UA: uric acid; BUN: blood urea nitrogen; P: phosphorus; LY: lymphocyte count; ALP: alkaline phosphatase; K: kalium; CK: creatine kinase; CREA: creatinine; AST: glutamic oxalacetic transaminase; ALT: glutamic-pyruvic transaminase; GGT: gamma-glutamyl transpeptidase; TG: triglyceride; CA: calcium; MONP: percentage of monocyte; APTT: activated partial thromboplastin time; INR: international normalized ratio; RBCSD: red blood cell distribution width-standard deviation; PTP: percentage of prothrombin time; MCV: mean corpuscular volume; MCHC: mean corpuscular hemoglobin concentration; DD: d-dimer; BASP: percentage of basophil; CL: chloridion; DBIL: direct bilirubin

Table S6. Compare baseline characteristics between HM and HMRD group in internal validation set

|  | HM | HMRD | P-value |
| --- | --- | --- | --- |
| N | 5694 | 1317 |  |
| AGE | 36.64 ± 15.96 | 51.50 ± 14.17 | <0.001 |
| GENDER |  |  | <0.001 |
| Male | 1571 (27.59%) | 688 (52.24%) |  |
| Female | 4123 (72.41%) | 629 (47.76%) |  |
| TBA | 3.50 ± 3.63 | 4.48 ± 4.41 | <0.001 |
| TBIL(μmol/L) | 11.02 ± 5.28 | 11.52 ± 5.36 | 0.002 |
| TC(mmol/L) | 4.53 ± 0.90 | 4.78 ± 1.01 | <0.001 |
| N(10^9/L) | 3.68 ± 1.32 | 3.95 ± 1.39 | <0.001 |
| ALB(g/L) | 48.15 ± 3.35 | 47.40 ± 3.21 | <0.001 |
| RBC(10^12/L) | 4.52 ± 0.45 | 4.64 ± 0.48 | <0.001 |
| HBA1C(%) | 5.36 ± 0.56 | 5.64 ± 0.79 | <0.001 |
| PCT(%) | 0.24 ± 0.05 | 0.22 ± 0.05 | <0.001 |
| PDW(%) | 12.32 ± 2.72 | 12.96 ± 2.92 | <0.001 |
| GLU(mmol/L) | 5.34 ± 1.07 | 6.00 ± 1.53 | <0.001 |
| FIB(g/L) | 2.74 ± 0.59 | 2.92 ± 0.71 | <0.001 |
| EOSP(%) | 1.77 ± 1.62 | 1.74 ± 1.76 | 0.563 |
| BASP(%) | 0.41 ± 0.27 | 0.35 ± 0.29 | <0.001 |
| LDH(U/L) | 155.38 ± 30.30 | 166.38 ± 32.63 | <0.001 |
| GLB(g/L) | 26.94 ± 4.19 | 28.11 ± 4.37 | <0.001 |
| PAB(mg/L) | 268.23 ± 52.03 | 295.92 ± 65.57 | <0.001 |
| MPV(fL) | 10.15 ± 1.12 | 10.20 ± 1.16 | 0.136 |
| TT(s) | 17.99 ± 1.43 | 17.94 ± 1.14 | 0.266 |
| UA(mmol/L) | 0.30 ± 0.07 | 0.31 ± 0.07 | <0.001 |
| BUN(mmol/L) | 4.71 ± 1.31 | 5.39 ± 1.65 | <0.001 |
| SODIUM(mmol/L) | 140.87 ± 2.05 | 141.65 ± 2.34 | <0.001 |
| CL(mmol/L) | 102.85 ± 2.28 | 102.87 ± 2.51 | 0.783 |
| P(mmol/L) | 1.18 ± 0.17 | 1.12 ± 0.18 | <0.001 |
| LY(10^9/L) | 1.89 ± 0.56 | 1.81 ± 0.87 | <0.001 |
| ALP(U/L) | 64.56 ± 20.18 | 76.16 ± 22.13 | <0.001 |
| K(mmol/L) | 4.22 ± 0.34 | 4.21 ± 0.38 | 0.637 |
| CK(U/L) | 113.31 ± 273.28 | 99.42 ± 71.96 | 0.067 |
| CREA(μmol/L) | 65.10 ± 14.77 | 70.48 ± 19.04 | <0.001 |
| AST(U/L) | 18.77 ± 9.28 | 20.59 ± 9.08 | <0.001 |
| ALT(U/L) | 17.69 ± 17.05 | 22.49 ± 20.58 | <0.001 |
| GGT(U/L) | 20.31 ± 23.67 | 27.70 ± 25.42 | <0.001 |
| TG(mmol/L) | 1.19 ± 1.01 | 1.68 ± 1.23 | <0.001 |
| CA(mmol/L) | 2.35 ± 0.10 | 2.35 ± 0.10 | 0.345 |
| MONP(%) | 6.40 ± 1.68 | 6.33 ± 1.71 | 0.157 |
| APTT(s) | 33.35 ± 5.71 | 33.24 ± 5.92 | 0.510 |
| INR | 0.97 ± 0.08 | 0.96 ± 0.07 | 0.119 |
| RBCSD(fL) | 40.58 ± 2.88 | 41.42 ± 3.08 | <0.001 |
| PTP(%) | 106.97 ± 12.81 | 108.88 ± 13.58 | <0.001 |
| MCV(fL) | 89.24 ± 4.87 | 90.11 ± 5.05 | <0.001 |
| MCHC(g/L) | 334.27 ± 11.59 | 336.16 ± 11.38 | <0.001 |
| DD(μg/mL) | 0.32 ± 2.28 | 18.19 ± 127.48 | <0.001 |
| DBIL(μmol/L) | 4.37 ± 1.79 | 4.18 ± 1.84 | <0.001 |

TBA: total bile acid; TBIL: total bilirubin; TC: total cholesterol; N: neutrophil number; ALB: albumin; RBC: red blood count; HBA1C: glycosylated hemoglobin; PCT: thrombocytocrit; PDW: platelet distribution width; GLU: glucose; FIB: fibrinogen; EOSP: percentage of eosinophils; GLB: globulin; PAB: prealbumin; MPV: mean platelet volume; TT: thrombin time; UA: uric acid; BUN: blood urea nitrogen; P: phosphorus; LY: lymphocyte count; ALP: alkaline phosphatase; K: kalium; CK: creatine kinase; CREA: creatinine; AST: glutamic oxalacetic transaminase; ALT: glutamic-pyruvic transaminase; GGT: gamma-glutamyl transpeptidase; TG: triglyceride; CA: calcium; MONP: percentage of monocyte; APTT: activated partial thromboplastin time; INR: international normalized ratio; RBCSD: red blood cell distribution width-standard deviation; PTP: percentage of prothrombin time; MCV: mean corpuscular volume; MCHC: mean corpuscular hemoglobin concentration; DD: d-dimer; BASP: percentage of basophil; CL: chloridion; DBIL: direct bilirubin

Table S7. Compare baseline characteristics between HM and HMRD group in external test set

|  | HM | HMRD | P-value |
| --- | --- | --- | --- |
| N | 1487 | 515 |  |
| AGE | 35.60 ± 16.69 | 47.31 ± 17.20 | <0.001 |
| GENDER |  |  | 0.124 |
| Male | 1302 (87.56%) | 464 (90.10%) |  |
| Female | 185 (12.44%) | 51 (9.90%) |  |
| TBA(μmol/L) | 3.71 ± 3.30 | 4.23 ± 3.54 | 0.003 |
| TBIL(μmol/L) | 13.10 ± 5.97 | 12.95 ± 6.03 | 0.618 |
| TC(mmol/L) | 4.74 ± 0.95 | 4.86 ± 1.01 | 0.013 |
| N(10^9/L) | 4.07 ± 1.36 | 4.26 ± 1.33 | 0.005 |
| ALB(g/L) | 49.48 ± 3.58 | 48.36 ± 3.58 | <0.001 |
| RBC(10^12/L) | 5.03 ± 0.49 | 4.91 ± 0.48 | <0.001 |
| HBA1C(%) | 5.50 ± 0.67 | 5.64 ± 0.73 | <0.001 |
| PCT(%) | 0.24 ± 0.05 | 0.23 ± 0.05 | <0.001 |
| PDW(%) | 12.53 ± 2.67 | 12.69 ± 2.94 | 0.261 |
| GLU(mmol/L) | 5.54 ± 1.17 | 5.82 ± 1.35 | <0.001 |
| FIB(g/L) | 2.74 ± 0.65 | 3.02 ± 0.78 | <0.001 |
| EOSP(%) | 2.10 ± 1.74 | 1.93 ± 1.54 | 0.046 |
| LDH(U/L) | 167.24 ± 38.50 | 170.59 ± 31.07 | 0.075 |
| GLB(g/L) | 27.23 ± 4.31 | 28.58 ± 4.49 | <0.001 |
| PAB(mg/L) | 322.97 ± 64.55 | 333.79 ± 80.71 | 0.002 |
| MPV(fL) | 10.13 ± 1.09 | 10.21 ± 1.20 | 0.157 |
| TT(s) | 18.51 ± 1.16 | 18.11 ± 1.13 | <0.001 |
| UA(mmol/L) | 0.51 ± 0.05 | 0.50 ± 0.05 | <0.001 |
| BUN(mmol/L) | 5.32 ± 1.55 | 5.83 ± 1.88 | <0.001 |
| P(mmol/L) | 1.16 ± 0.18 | 1.14 ± 0.18 | 0.028 |
| LY(10^9/L) | 2.07 ± 0.64 | 1.97 ± 0.64 | 0.002 |
| ALP(U/L) | 75.02 ± 23.95 | 77.56 ± 20.52 | 0.032 |
| K(mmol/L) | 4.28 ± 0.36 | 4.28 ± 0.39 | 0.747 |
| CK(U/L) | 172.70 ± 306.10 | 120.19 ± 73.96 | <0.001 |
| CREA(μmol/L) | 84.82 ± 18.57 | 89.42 ± 29.48 | <0.001 |
| AST(U/L) | 25.48 ± 20.52 | 25.29 ± 13.34 | 0.847 |
| ALT(U/L) | 36.57 ± 31.66 | 35.76 ± 31.69 | 0.614 |
| GGT(U/L) | 41.97 ± 51.58 | 43.40 ± 38.54 | 0.566 |
| TG(mmol/L) | 1.95 ± 1.47 | 2.35 ± 1.96 | <0.001 |
| CA(mmol/L) | 2.41 ± 0.11 | 2.39 ± 0.12 | 0.002 |
| MONP(%) | 6.84 ± 1.70 | 6.67 ± 1.77 | 0.053 |
| APTT(s) | 32.43 ± 6.09 | 33.63 ± 5.64 | <0.001 |
| INR | 0.95 ± 0.07 | 0.96 ± 0.10 | 0.040 |
| RBCSD(fL) | 39.86 ± 2.81 | 40.93 ± 3.22 | <0.001 |
| PTP(%) | 108.75 ± 13.46 | 108.87 ± 13.38 | 0.862 |
| MCV(fL) | 88.44 ± 4.50 | 89.56 ± 4.30 | <0.001 |
| MCHC(g/L) | 339.45 ± 11.29 | 340.63 ± 10.52 | 0.038 |
| DD(μg/mL) | 0.48 ± 8.68 | 13.74 ± 107.99 | <0.001 |
| BASP(%) | 0.41 ± 0.25 | 0.37 ± 0.26 | 0.001 |
| SODIUM(mmol/L) | 141.40 ± 2.04 | 141.87 ± 2.28 | <0.001 |
| CL(mmol/L) | 102.20 ± 2.49 | 102.62 ± 2.81 | 0.002 |
| DBIL(μmol/L) | 5.03 ± 1.94 | 4.70 ± 2.03 | <0.001 |

TBA: total bile acid; TBIL: total bilirubin; TC: total cholesterol; N: neutrophil number; ALB: albumin; RBC: red blood count; HBA1C: glycosylated hemoglobin; PCT: thrombocytocrit; PDW: platelet distribution width; GLU: glucose; FIB: fibrinogen; EOSP: percentage of eosinophils; GLB: globulin; PAB: prealbumin; MPV: mean platelet volume; TT: thrombin time; UA: uric acid; BUN: blood urea nitrogen; P: phosphorus; LY: lymphocyte count; ALP: alkaline phosphatase; K: kalium; CK: creatine kinase; CREA: creatinine; AST: glutamic oxalacetic transaminase; ALT: glutamic-pyruvic transaminase; GGT: gamma-glutamyl transpeptidase; TG: triglyceride; CA: calcium; MONP: percentage of monocyte; APTT: activated partial thromboplastin time; INR: international normalized ratio; RBCSD: red blood cell distribution width-standard deviation; PTP: percentage of prothrombin time; MCV: mean corpuscular volume; MCHC: mean corpuscular hemoglobin concentration; DD: d-dimer; BASP: percentage of basophil; CL: chloridion; DBIL: direct bilirubin

Table S8. The model performance of the random sampling 1 set

| Model | Sensitivity | Specificity | Accuracy | AUC | AUCPR | PPV | NPV | Balanced accuracy |
| --- | --- | --- | --- | --- | --- | --- | --- | --- |
| Train |  |  |  |  |  |  |  |  |
| RF | 0.9984 | 0.9627 | 0.9823 | 0.9995 | 0.9997 | 0.9701 | 0.9980 | 0.9805 |
| GBM | 0.9075 | 0.6401 | 0.7868 | 0.8665 | 0.8749 | 0.7540 | 0.8507 | 0.7738 |
| GLM | 0.8829 | 0.6259 | 0.7669 | 0.8117 | 0.7930 | 0.7415 | 0.8148 | 0.7544 |
| DL | 0.9152 | 0.5892 | 0.7681 | 0.8298 | 0.8299 | 0.7303 | 0.8512 | 0.7522 |
| Validate |  |  |  |  |  |  |  |  |
| RF | 0.8785 | 0.6253 | 0.7644 | 0.8172 | 0.8085 | 0.7407 | 0.8086 | 0.7519 |
| GBM | 0.8975 | 0.6087 | 0.7673 | 0.8339 | 0.8412 | 0.7364 | 0.8298 | 0.7531 |
| GLM | 0.8724 | 0.6087 | 0.7535 | 0.7954 | 0.7815 | 0.7309 | 0.7966 | 0.7406 |
| DL | 0.9036 | 0.5717 | 0.7540 | 0.8047 | 0.8030 | 0.7199 | 0.8295 | 0.7376 |
| Test |  |  |  |  |  |  |  |  |
| RF | 0.7146 | 0.5907 | 0.6615 | 0.7076 | 0.7490 | 0.6996 | 0.6080 | 0.6526 |
| GBM | 0.7398 | 0.6192 | 0.6881 | 0.7491 | 0.7967 | 0.7216 | 0.6408 | 0.6795 |
| GLM | 0.8330 | 0.4689 | 0.6770 | 0.7264 | 0.7524 | 0.6767 | 0.6779 | 0.6510 |
| DL | 0.3165 | 0.8782 | 0.5572 | 0.6335 | 0.7133 | 0.7762 | 0.4906 | 0.5974 |

Table S9. The model performance of the random sampling 2 set

| Model | Sensitivity | Specificity | Accuracy | AUC | AUCPR | PPV | NPV | Balanced accuracy |
| --- | --- | --- | --- | --- | --- | --- | --- | --- |
| Train |  |  |  |  |  |  |  |  |
| RF | 0.9966 | 0.9768 | 0.9876 | 0.9997 | 0.9998 | 0.9812 | 0.9957 | 0.9867 |
| GBM | 0.9319 | 0.6681 | 0.8128 | 0.9113 | 0.9246 | 0.7734 | 0.8898 | 0.8000 |
| GLM | 0.8967 | 0.6140 | 0.7691 | 0.8164 | 0.7993 | 0.7384 | 0.8302 | 0.7553 |
| DL | 0.9060 | 0.6056 | 0.7704 | 0.8405 | 0.8462 | 0.7363 | 0.8412 | 0.7558 |
| Validate |  |  |  |  |  |  |  |  |
| RF | 0.8709 | 0.6438 | 0.7686 | 0.8313 | 0.8351 | 0.7487 | 0.8037 | 0.7574 |
| GBM | 0.8998 | 0.6207 | 0.7740 | 0.8461 | 0.8564 | 0.7429 | 0.8356 | 0.7602 |
| GLM | 0.8800 | 0.6087 | 0.7577 | 0.8041 | 0.7796 | 0.7326 | 0.8064 | 0.7444 |
| DL | 0.8937 | 0.5976 | 0.7602 | 0.8201 | 0.8126 | 0.7301 | 0.8219 | 0.7456 |
| Test |  |  |  |  |  |  |  |  |
| RF | 0.7282 | 0.6399 | 0.6903 | 0.7498 | 0.7907 | 0.7296 | 0.6382 | 0.6840 |
| GBM | 0.7282 | 0.6554 | 0.6970 | 0.7516 | 0.7923 | 0.7382 | 0.6438 | 0.6918 |
| GLM | 0.8583 | 0.4585 | 0.6870 | 0.7401 | 0.7637 | 0.6790 | 0.7080 | 0.6584 |
| DL | 0.2233 | 0.9467 | 0.5461 | 0.6838 | 0.7704 | 0.9274 | 0.4852 | 0.6000 |

Table S10. The model performance of the random sampling 3 set

| Model | Sensitivity | Specificity | Accuracy | AUC | AUCPR | PPV | NPV | Balanced accuracy |
| --- | --- | --- | --- | --- | --- | --- | --- | --- |
| Train |  |  |  |  |  |  |  |  |
| RF | 0.9989 | 0.9514 | 0.9775 | 0.9994 | 0.9997 | 0.9615 | 0.9986 | 0.9752 |
| GBM | 0.9608 | 0.6697 | 0.8294 | 0.9426 | 0.9515 | 0.7795 | 0.9336 | 0.8152 |
| GLM | 0.9105 | 0.5683 | 0.7560 | 0.8045 | 0.7819 | 0.7193 | 0.8393 | 0.7394 |
| DL | 0.8747 | 0.6587 | 0.7772 | 0.8275 | 0.8256 | 0.7570 | 0.8122 | 0.7667 |
| Validate |  |  |  |  |  |  |  |  |
| RF | 0.8945 | 0.6300 | 0.7752 | 0.8301 | 0.8301 | 0.7465 | 0.8305 | 0.7622 |
| GBM | 0.9051 | 0.6189 | 0.7761 | 0.8453 | 0.8508 | 0.7431 | 0.8426 | 0.7620 |
| GLM | 0.8967 | 0.5920 | 0.7594 | 0.8055 | 0.7783 | 0.7281 | 0.8247 | 0.7444 |
| DL | 0.8527 | 0.6725 | 0.7715 | 0.8124 | 0.8046 | 0.7603 | 0.7894 | 0.7626 |
| Test |  |  |  |  |  |  |  |  |
| RF | 0.6738 | 0.6425 | 0.6604 | 0.7075 | 0.7384 | 0.7155 | 0.5962 | 0.6581 |
| GBM | 0.3942 | 0.9016 | 0.6115 | 0.7604 | 0.7978 | 0.8423 | 0.5273 | 0.6479 |
| GLM | 0.8350 | 0.4508 | 0.6704 | 0.7324 | 0.7537 | 0.6698 | 0.6718 | 0.6429 |
| DL | 0.0680 | 0.9948 | 0.4650 | 0.6378 | 0.7282 | 0.9459 | 0.4444 | 0.5314 |

Table S11. The relationship between nine features and RD by Spearman analysis

|  | Male | Older age | PCT | GLU | BASP | GLB | MPV | UA | APTT |
| --- | --- | --- | --- | --- | --- | --- | --- | --- | --- |
| r | -0.206 | 0.3388 | -0.117 | 0.2435 | -0.109 | 0.1086 | 0.0223 | 0.074 | -0.02 |
| P | <0.001 | <0.001 | <0.001 | <0.001 | <0.001 | <0.001 | <0.001 | <0.001 | <0.001 |

Table S12. The relationship between nine features and RD by Logistic regression analysis

|  | OR | 95%CI | P value |
| --- | --- | --- | --- |
| Age | 1.05 | 1.05, 1.05 | <0.0001 |
| Gender |  |  |  |
| Female | 1.0 |  |  |
| Male | 2.85 | 2.07, 3.56 | <0.0001 |
| PCT | 0.01 | 0.01, 0.01 | <0.0001 |
| GLU | 1.39 | 1.36, 1.42 | <0.0001 |
| BASP | 0.34 | 0.30, 0.39 | <0.0001 |
| GLB | 1.07 | 1.06, 1.07 | <0.0001 |
| MPV | 1.07 | 1.04, 1.10 | <0.0001 |
| UA | 6.89 | 4.97, 9.55 | <0.0001 |
| APTT | 0.65 | 0.48, 0.82 | <0.0001 |

**Figure S1.** Relationship among age, APTT, BASP, gender, GLB, GLU, MPV, PCT, and UA

**Figure S2.** Random sample set 1. The area under the receiver operating characteristic curve (AUC) of the random forest (A), GBM (B), GLM (C), and deep learning (D) models based on the nine selected variables in the training set, internal validation set and the external test set. The area under the precision-recall curve (AUCPR) of the random forest (E), GBM (F), GLM (G), and deep learning (H) models based on the nine selected variables in the training set, internal validation set and the external test set.

**Figure S3.** Random sample set 2. The area under the receiver operating characteristic curve (AUC) of the random forest (A), GBM (B), GLM (C), and deep learning (D) models based on the nine selected variables in the training set, internal validation set and the external test set. The area under the precision-recall curve (AUCPR) of the random forest (E), GBM (F), GLM (G), and deep learning (H) models based on the nine selected variables in the training set, internal validation set and the external test set.

**Figure S4.** Random sample set 3. The area under the receiver operating characteristic curve (AUC) of the random forest (A), GBM (B), GLM (C), and deep learning (D) models based on the nine selected variables in the training set, internal validation set and the external test set. The area under the precision-recall curve (AUCPR) of the random forest (E), GBM (F), GLM (G), and deep learning (H) models based on the nine selected variables in the training set, internal validation set and the external test set.

**Ophthalmic and medical examinations**

The examinations included slit-lamp examination, uncorrected distance visual acuity, corrected distance visual acuity, autorefraction, manifest refraction, intraocular pressure (IOP), and funduscopic examinations. Wide-angle fundus photograph was performed. The central corneal thickness, axial length, and anterior chamber depth were all measured using an A-scan ultrasonography (A-Scan Pachymeter, Ultrasonic, Exton, PA, USA).

**Inclusion and exclusion criteria**

Emmetropia was defined as a mean spherical equivalent (SE) ranging from −0.25 to +0.25 diopters (D). High myopia was defined as a SE of ≤ −6.00 D.

Inclusion criteria of HM: (1) age ≧ 18 years; (2) SE of − 6.00 D or higher; (3) the age of HM diagnosis less than 40 years. Exclusion criteria of HM: (1) missing refraction data; (2) history of fundus oculi surgery/barrier laser and self-reported refractive surgery; (3) retinal detachment; (4) other types of fundus oculi diseases, such as macular degeneration, diabetic retinopathy, glaucoma and so on; (5) ocular trauma; (6) coagulation disorders; (7) hematologic diseases; (8) received drugs that can affect blood components; (9) systemic diseases, such as infectious diseases, metabolic syndrome, autoimmune disorders, and cancer.

Inclusion criteria of HMRD: (1) age ≧ 18 years; (2) SE of − 6.00 D or higher; (3) retinal detachment; (4) the age of HM diagnosis less than 40 years. Exclusion criteria of HMRD: (1) missing refraction data; (2) history of fundus oculi surgery/barrier laser and self-reported refractive surgery; (3) other types of fundus oculi diseases, such as macular degeneration, diabetic retinopathy, glaucoma and so on; (4) ocular trauma; (5) coagulation disorders; (6) hematologic diseases; (7) received drugs that can affect blood components; (8) systemic diseases, including acute infectious diseases, metabolic syndrome, autoimmune disease, and cancer.
